# Time-lapse electrical impedance spectroscopy for monitoring the cell cycle of single immobilized *S. pombe* cells

**DOI:** 10.1038/srep17180

**Published:** 2015-11-26

**Authors:** Zhen Zhu, Olivier Frey, Niels Haandbaek, Felix Franke, Fabian Rudolf, Andreas Hierlemann

**Affiliations:** 1 ETH Zurich, Department of Biosystems Science and Engineering, Bio Engineering Laboratory, Mattenstrasse 26, CH-4058 Basel, Switzerland; 2 ETH Zurich, Department of Biosystems Science and Engineering, Computational Systems Biology Group, Mattenstrasse 26, CH-4058 Basel, Switzerland

## Abstract

As a complement and alternative to optical methods, wide-band electrical impedance spectroscopy (EIS) enables multi-parameter, label-free and real-time detection of cellular and subcellular features. We report on a microfluidics-based system designed to reliably capture single rod-shaped *Schizosaccharomyces pombe* cells by applying suction through orifices in a channel wall. The system enables subsequent culturing of immobilized cells in an upright position, while dynamic changes in cell-cycle state and morphology were continuously monitored through EIS over a broad frequency range. Besides measuring cell growth, clear impedance signals for nuclear division have been obtained. The EIS system has been characterized with respect to sensitivity and detection limits. The spatial resolution in measuring cell length was 0.25 μm, which corresponds to approximately a 5-min interval of cell growth under standard conditions. The comprehensive impedance data sets were also used to determine the occurrence of nuclear division and cytokinesis. The obtained results have been validated through concurrent confocal imaging and plausibilized through comparison with finite-element modeling data. The possibility to monitor cellular and intracellular features of single *S. pombe* cells during the cell cycle at high spatiotemporal resolution renders the presented microfluidics-based EIS system a suitable tool for dynamic single-cell investigations.

Continuous single-cell analysis with high-spatiotemporal resolution offers the potential to obtain information on factors that influence cellular heterogeneity in biological systems^1^−^3^. The most common method for monitoring cellular dynamics at single-cell resolution is optical microscopy. The development of genetically encoded fluorophores and non-toxic chemical fluorophores made it possible to simultaneously monitor multiple processes in a single cell over extended times^4^.

Optical and fluorescence methods are widely used and established, however, they require the use of fluorescence labels, which may interfere with protein function and thereby impact cellular development^5^,^6^. Moreover, the application of intense light needed to excite the fluorophores may release or produce toxic compounds in the cells, which then may upset or interfere with the naturally occurring processes in living cells^7^,^8^. Label-free methods could overcome these limitations but they often lack the resolution of optical microscopy. Therefore, a combination of fluorescence microscopy with label-free methods, which provide additional information on a biological process, is desirable.

A candidate method that can provide complementary information on cellular and subcellular properties is electrical impedance spectroscopy (EIS)^9^. EIS relies on applying an external field of variable frequency to measure the dielectric properties of a sample that interacts with that external field, while the sample is usually placed between electrodes or within the electric field^9^,^10^. Two different parameters are usually measured, the impedance magnitude, which is the ratio of the amplitude of the applied voltage to the amplitude of the measured current, and the phase, i.e., the phase shift by which the current lags behind the voltage. EIS is non-invasive and label-free and has been used to analyze the dielectric properties of particles and biological cells^9^,^10^. Depending on the frequency of the applied electric field, different information on the probed cells can be extracted^11^. At low frequencies between ~100 kHz and ~1 MHz, information on the cell size and volume can be obtained. At higher frequencies, above 1 MHz information related to the cell membrane (open ion channels, membrane polarization) and information on intracellular compartments, such as cytoplasm, vacuoles, and the cell nucleus, can be gained. Impedance spectroscopy can also be used to detect cell motion^12^,^13^ or cellular mechanical (muscle cells)^14^ and electrical (cardiac cells) activity^15^.

Several groups performed EIS-based cell characterizations by means of microfluidic devices^11^,^16^. The majority of them implemented EIS in continuous-flow systems^17^−^20^. In analogy to flow cytometry, these systems allow for rapid multi-parameter analysis of large numbers of single cells, which can be classified according to cell size and dielectric properties. The resulting data, however, include recordings at single time points so that continuous monitoring of selected cells is impossible, as is the assignment of time-lapse signals to the respective cells.

For extended-time monitoring of single cells, these cells need to be individually trapped under precisely controlled culturing conditions by dedicated microstructures^21^−^23^ that contain electrodes. One of the most popular cell immobilization methods relies on microwell arrays to trap single cells by sedimentation^24^−^26^. Another frequently utilized approach is to passively capture single cells with specially designed microstructures by using hydrodynamic forces^27^−^30^, where, however, the capture of cells relies on stochastic processes so that it is impossible to select cells of interest and to then precisely control the immobilization and retention of these selected individual cells over extended times. More details on the immobilization requirements will be given in the Results section.

EIS measurements then have to be continuously performed on the immobilized cells at the traps by means of electrodes. Experimental evidence presented to date includes comparisons of the signal magnitude before and after trapping of a single cell^31^,^32^, or the variation of impedance signals upon perfusing different media over the trapped cells^33^. Another approach is to seed cells directly on large electrode-covered surfaces to detect impedance magnitude and phase changes upon culturing of a cell population over a longer time period. The signal changes then can be correlated with initial cell density and cell growth or proliferation, with cell vitality (dead or alive also upon dosage of compounds), as well as with cellular processes, such as cell-substrate interactions, cell attachment and cell motility^12^,^34^−^37^.

*Schizosaccharomyces pombe* is a frequently-used model organism to study cell growth and cell cycle^38^−^40^. It has a characteristic rod-shape structure with sturdy cell walls, is straightforward to cultivate in microfluidic devices, and features a defined cell cycle with temporally separated mitosis and cytokinesis (Fig. 1a). A strain bearing a fluorescent protein, which inserts in the endoplasmic reticulum (ER) membrane and thereby helps to visualize the cell and nuclear boundaries, has been used to enable concurrent confocal imaging, which was performed every 20 minutes to avoid bleaching and phototoxic effects.

A typical cell cycle of *S. pombe* comprises four phases, a long G2, M (mitotic), a short G1 and S (synthesis) phase^38^. Figure 1a shows fluorescence micrographs of an *S. pombe* cell at different cell-cycle states (S1–S8). Corresponding growth curves, measured by optical imaging, are plotted in Fig. 1b. In the G2 phase, an *S. pombe* cell grows exclusively through the cell tips: growth starts at a cell length of around 8 μm and proceeds to around 14 μm by elongation of both ends (S1–S4). Once a critical size is reached, cell elongation slows down and a septum forms in the middle of the cell. Around the time at which growth in length stops, cells enter the M phase observable by elongation of the nucleus (S5) and subsequent division into two nuclei (S6). Ultimately, the cell splits and produces two distinct daughter cells of equal size (S8). It is important to notice that the cytokinesis of *S. pombe* occurs after the G1 phase and around the time DNA synthesis and chromosome duplication (S phase) are completed (S7) or even early in the G2 phase.

The central goal of our work was to use continuous EIS multi-frequency monitoring of single cells to detect developmental changes during the cell cycle, such as the process of nuclear division during mitosis and cytokinesis. In particular, we were interested in sensitivity, resolution, and the capability to detect dynamic cellular processes, as EIS has the potential to complement optical microscopy for *in situ* single-cell analysis. We have previously shown that differences in intracellular features, such as the presence of small and large vacuoles in yeast cells, can be detected by EIS in flow-through-mode by using frequencies up to 200 MHz^41^. Continuous monitoring of developmental changes including nuclear division of single-cells has, however, to the best of our knowledge, not been demonstrated up to date.

We characterized the device by using monodisperse polystyrene (PS) beads to assess EIS sensitivity and detection limits: stacks of different numbers and sizes of beads were used to simulate cells of different lengths, and concurrent impedance signals were recorded. As beads do not have any features and do not undergo any changes over time, their signals over all used frequencies can be used as a reference. We then performed real-time EIS monitoring of the life cycle of individual *S. pombe* cells at a high resolution. We could monitor cell growth at a resolution of 0.25 μm, and we could clearly identify intracellular events from their impedance signatures. We were able to distinguish cells undergoing nuclear division from cells that did not divide. As the *S. pombe* strain featured the fluorescent protein in the endoplasmic reticulum (ER), concurrent confocal fluorescence imaging could be used to detect the cell and nuclear boundaries. Imaging was performed at an interval of 20 minutes to avoid bleaching and phototoxic effects. The impedance results have been validated with the acquired confocal images and plausibilized through finite-element modeling.

## Results

### Cell immobilization and EIS measurements

The utilized microfluidics-based EIS microsystem must enable cell cultivation for at least 2-3 hours and provide stable cell immobilization for the continuous multi-frequency single-cell impedance measurements over a complete cell cycle, as already minute position variations will lead to large impedance changes. It needs a controllable trap-and-release function to capture target cells from the solution and, thereafter, release them for subsequent analysis or measuring the next cell. The system must also be amenable to high-resolution wide-field and confocal imaging in order to enable correlation and validation of the impedance data with simultaneously acquired optical images of the cells.

**Figure 1:**
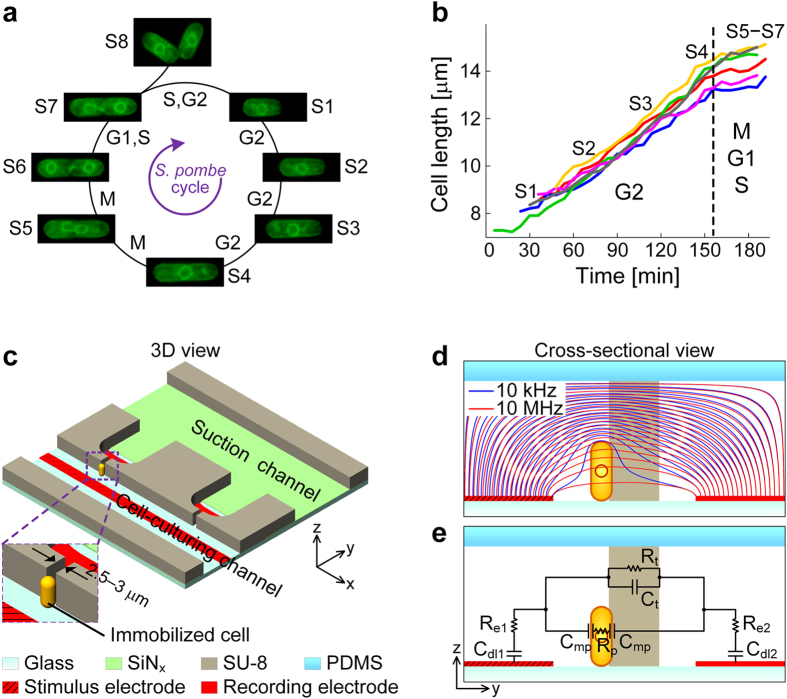
Cell cycle of *S. pombe* and microfluidic EIS device for cell immobilization and impedance recording. (**a**) Micrographs of an *S. pombe* cell during its cell cycle indicating the different processes occurring in the cell and the nucleus. (**b**) Growth curves of six *S. pombe* cells. Cells have been cultured on a glass slide and monitored by using time-lapse fluorescence imaging at an interval of 6 min. (**c**) Schematic 3D close-up of an immobilized *S. pombe* cell at the orifice of one trap located between cell-culturing and suction channels. The PDMS cover is not shown for better visibility. Electrodes on the bottom are indicated in red (see Methods section for detailed description). (**d**) Simulated current distribution across a trap with an immobilized *S. pombe* cell at low and high frequencies (10 kHz, 10 MHz). (**e**) Equivalent circuit model (ECM) components of an *S. pombe* cell immobilized at a trap. 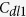, 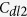, 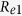, and 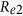 are the electrical double-layer capacitances and resistances of both microelectrodes, respectively; 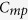 and 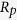 are the membrane capacitance and cellular resistance of the *S. pombe* cell; 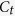 and 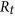 are the capacitance and resistance of the bulk medium across the trap.

Accordingly, we designed a microsystem, based on a predecessor single-cell cultivation device^13^,^42^, for fission yeast cells, *S. pombe*, which were immobilized in an upright “standing” position along the channel walls in front of orifices with the bottom end resting on the channel floor (see Fig. 1c,d). The immobilization of *S. pombe* is mechanically stable and offers the advantage of having a defined initial position from which the cells then grow in length and divide. Another advantage of the chosen cell orientation in the device is that the characteristic longitudinal cell growth and cell division of *S. pombe* occur in a plane, which is perpendicular to the electrical field lines arising from the electrodes, which brings about large EIS sensitivity, i.e., large impedance changes at different frequencies (Fig. 1d).

The microfluidic arrangement of the microdevice included a cell-culturing channel (150 μm width), a suction channel (300 μm width), and ten bottleneck-like orifices, located between the two channels, at which target cells can be trapped by applying underpressure in the suction channel. To ensure an active capturing and a reliable retention of single beads and cells, the device design implemented the following features and mechanisms: (i) narrow orifices (width of 2.5−3 μm, less than the diameter of the beads and *S. pombe* cells) enabled accurate and reliable immobilization through hydrodynamic forces; (ii) the liquid flows included pure medium, coming from the upper-left inlet and flowing along the trapping sites in the upper half of the cell-culturing channel, and the liquid in which the sample was suspended coming from the lower-left inlet and flowing along the lower half of the channel (Supplementary Figs S1a,b, S2); this flow arrangement kept the sample stream away from the traps during cell recordings; (iii) the parallel flow profile could be modulated by adjusting the pressure applied to the suction channel.

To capture a bead or cell, the input stream was directed towards the trap by applying a lower pressure (relative to the pressure in the cell-culturing channel) to the suction channel. Once a bead or cell had been immobilized, the applied pressure was elevated immediately to an optimized value of 500 Pa (lower than the pressure in the cell-culturing channel) to restore parallel media and or particle streams. The immobilized bead or cell could, therefore, be reliably retained without risk to lose it or to capture additional particles at the trap. Computational fluid dynamics (CFD) simulations schematically illustrate the variation of the flow streams during this procedure of capturing and retaining a single particle at a trap (Supplementary Fig. S2). The trap geometry facilitated vertical *S. pombe* immobilization (“stand-up” mode) by hydrodynamic forces (Supplementary Movie S1), which entailed a defined initial position for each cell with the cell bottom standing on the channel floor.

For conducting EIS, a common microelectrode, which served as the stimulus electrode, was located along the length of the cell-culturing channel, and individual microelectrodes for signal recording were situated at each trapping site (Supplementary Fig. S1a,b). The planar microelectrodes on both sides of the orifices produced a horizontal distribution of the electric field lines (Fig. 1d). The narrow orifices of the traps condensed the electric field and constrained the electric current flow between stimulus and recording electrode. The orthogonal orientation of *S. pombe* with regard to the electric field allowed for sensitive monitoring of cell growth and cell division processes with high sensitivity (Supplementary Movie S2). Wide-band EIS has been implemented to broadly screen and measure cellular properties. Simulated field lines in Fig. 1d illustrate that, at low frequency (10 kHz), the electric current flows around the immobilized cell, while, at a higher frequency (10 MHz), the current penetrates the cell walls and plasma membranes, as the capacitive characteristics of the cell membrane vanish (at high frequencies the unloading of a large capacitor already starts before it is loaded to full capacity so that the capacitor behaves like a low-Ohmic resistor) and features inside the cell impact the impedance spectra. Equivalent-circuit model (ECM) components that have been used for modeling EIS signals of an immobilized *S. pombe* cell are shown in Fig. 1e. Details of the ECM are given in the Methods section.

Generally two impedance parameters of every immobilized cell or particle have been measured at 92 different frequencies between 10 kHz and 10 MHz, the relative magnitude, 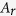, and the relative phase, 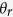. “Relative” means that the respective values for the empty trap have been subtracted, for details, see Methods section on electrical impedance spectroscopy.

### Sensitivity of EIS

EIS performance has been characterized by measuring beads with known diameters (Supplementary Note S1). We obtained strong correlations between the relative magnitudes 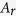 at 1 MHz and the heights of individual and stacked beads of different diameters. The spatial resolution of our system was determined to be 0.25 μm. The large sensitivity to height or height changes of immobilized samples can be attributed to the measurement configuration: a narrow orifice and an electric field oriented perpendicularly to the measured changes. The acquired impedance spectra over the whole frequency range show maximal variations of the relative magnitudes, 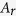, at 1 MHz, and conspicuous differences of the relative phase, 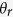, above 5 MHz (Supplementary Figs S5–7). We, therefore, selected these two frequencies of maximal variations for correlation of EIS measurements with corresponding cell cycle stages. It is important to note that, in the calibration measurements, the 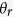 values at 5 MHz showed a rigorously linear correlation to the 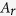 values at 1 MHz, which can be expected for invariable plastic beads.

### Measuring cell length through EIS

**Figure 2:**
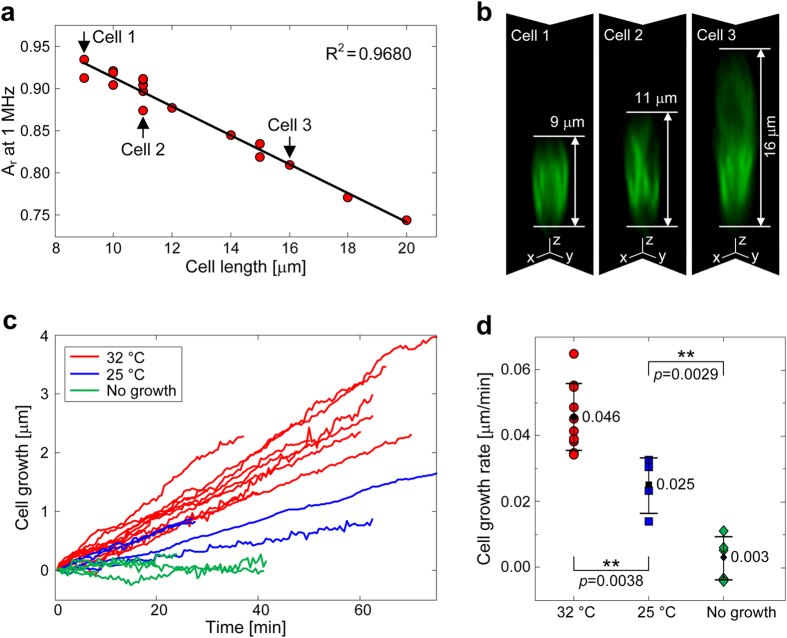
Length and growth measurements of *S. pombe* cells using EIS and confocal imaging. (**a**) Linear relationship between cell length measured by optical images and corresponding relative magnitude signals at 1 MHz of EIS. (**b**) Fluorescence micrographs of three exemplary cells represented as xz- and yz-plane intersections of the cells. (**c**) Growth recording (G2 phase) of immobilized *S. pombe* cells at different temperatures using EIS. Recorded relative cell growth curves at 32 °C (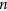 = 10) and 25 °C (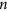 = 4) over different time periods. Five cells did not grow at all. (**d**) Determined cell growth rates and respective average values.

Calibration measurements of *S. pombe* cells bearing an endogenous Erg11-GFP fusion to fluorescently label the endoplasmic reticulum^43^ were performed by EIS together with confocal imaging to correlate the results of the two independent measurement methods. To this end, cells were trapped at the orifices, and EIS spectra as well as confocal-image z-stacks were acquired. Calibration measurements of the empty traps before and after cell immobilization were conducted for each device. Figure 2a shows a linear correlation (coefficient of determination, 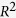 = 0.9680) between cell length measured by optical images, and relative impedance magnitudes, 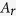, at 1 MHz. The exemplary fluorescence micrographs in Fig. 2b show three vertical confocal images of single cells (xz-plane and yz-plane intersections of each cell) with different heights. One can see that the upper half of the cells is not so trivial to resolve due to detection limits of the confocal microscope. These limitations rendered an automated image evaluation difficult so that the cell lengths had to be determined manually. The lowest limit of quantification (LLOQ) in the confocal micrographs was 0.5 μm, which is larger than the calculated LLOQ of the EIS measurements. Better linearity in determining the cell lengths may be achieved by making thinner slices during confocal imaging. However, photobleaching of fluorescent cells will then be faster, which would render a length determination again more difficult.

### Monitoring cell growth by using EIS

*S. pombe* cells spend most of their lifetime in interphases, especially in the G2 phase, growing by elongation at the ends. We monitored the immobilized *S. pombe* cells growing at two different temperatures, 32 °C and 25 °C. The change in the cell length over the recorded time period was extracted by referencing to the performed calibration measurements. In Fig. 2c, one can clearly identify the expected linearity of cell growth of *S. pombe* resulting from the continuous length elongation in G2 phase until a critical size was reached upon which polar growth ceased almost completely. Further, the expected different growth rates were observed for the two different temperatures. The extracted growth rate at 32 °C is 0.046 ± 0.010 μm min^−1^ (Fig. 2d), which compares well to published data (0.042 ± 0.055 μm min^−1^ of wild-type cells^40^,^43^) and is similar to the measured growth rate of 0.045 ± 0.005 μm min^−1^ at the same temperature on a glass slide using time-lapse imaging (Fig. 1b). The lower temperature reduced the growth rate to 0.025 ± 0.007 μm min^−1^, which has also been observed in conventional cultures 0.031 μm min^−1^ 43.

These results demonstrate that the EIS system exhibits enough sensitivity to enable continuous monitoring of the growth of single *S. pombe* cells. Importantly, the growth rates were found to be very similar for the different culturing methods and, therefore, it can be said that growth rates have neither been altered by the hydrodynamic immobilization, which retains the cell in a stand-up position, nor through phototoxic effects as a consequence of confocal imaging. The z-axis (height) resolution of the EIS system of 0.25 μm, corresponds to about 5 min growth time intervals, which can be resolved. We did not observe variations or drift in the impedance values of the reference measurements of the empty trap before and after a cell recording, so that any signal variation can be assigned to cell growth.

### Monitoring nuclear and cell division by using EIS

After a linear growth of the cell in the G2 phase, *S. pombe* enters the mitotic (M) phase. Cell growth slows down, and an intranuclear spindle is formed, which initiates nuclear division. The end of nuclear division or mitosis is indicated by the occurrence of two nuclei at the opposite ends of the cell. Subsequently, a septum in the middle of the cell is formed to separate the cell into two daughter cells (cytokinesis). Cytokinesis is the last step, where the final split from one cell into two daughter cells occurs. Mitosis in the *S. pombe* cell division cycle takes usually between 5 and 15 minutes. At 32 °C it takes between 20 and 45 minutes for a cell from stopping growth at its poles until separation into two cells.

We monitored the cell cycles of several immobilized *S. pombe* cells by using real-time EIS at an interval of 30 seconds, while we simultaneously imaged the very same cells with a confocal microscope every 20 min. The trapped cells are in different phases of their cycle and vary in their initial cell size, as we did not synchronize the cell cycle before the experiment. Therefore, the growth duration and time until the occurrence of mitosis varied.

**Figure 3:**
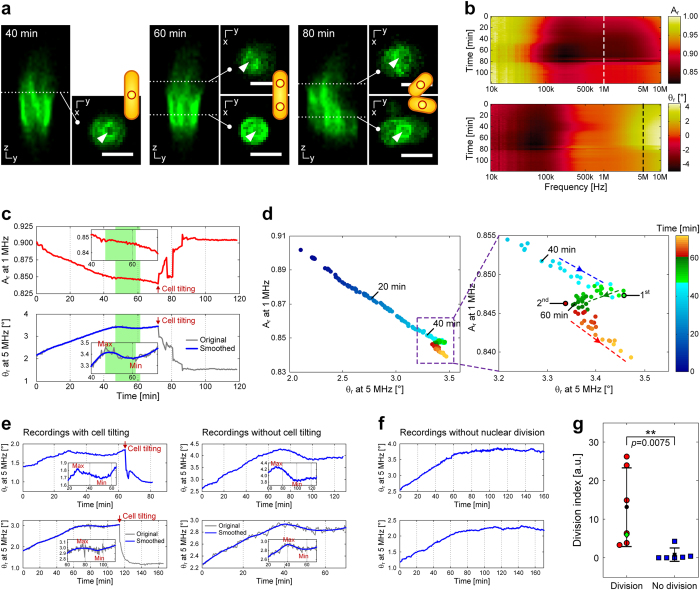
Monitoring nuclear division and cytokinesis of *S. pombe* cells monitored by using EIS. Fig. 3a–d show optical and EIS results for one exemplary single *S. pombe* cell, whereas Fig. 3e–g show EIS recordings and analysis results of additional cells. (**a**) Time-lapse confocal micrographs and corresponding cell cartoons at 40, 60 and 80 min after trapping of the cell. (**b**) Recorded EIS signals over the whole frequency range from 10 kHz to 10 MHz, plotted as relative magnitude and relative phase signals with respect to empty-trap reference signals (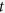 = 0 min represents the start of the respective experiment). (**c**) Growth curve displayed as 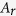 at 1 MHz and 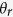 at 5 MHz versus time, respectively. Green-shaded area indicates the time period of nuclear and cell division. The red arrows indicate the moment when the tilting of daughter cells occurred. (**d**) Plot of 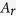 at 1 MHz versus 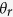 at 5 MHz during cell growth, nuclear division and cytokinesis. The close-up view indicates the trends in the correlation and the resulting turning points (1^st^ and 2^nd^). (**e**) Phase curves obtained from EIS recordings of four additional cells showing nuclear division (two recordings at the left: cell tilting after division; two recordings at the right: no cell tilting after division). The cells have not been synchronized so that recording starts at different time points in the growth phase, but always before mitosis, the time from maximum to minimum is always around 20 minutes. (**f**) Phase curves obtained from EIS recordings of two other cells not undergoing nuclear division. (**g**) The presence of nuclear division can be detected directly in the EIS phase signals. The *division index* computed from the EIS phase signals (see Methods) is significantly higher for the condition of “cell division” (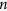 = 6) than for the condition of “no cell division” (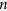 = 6). The diamond symbol (
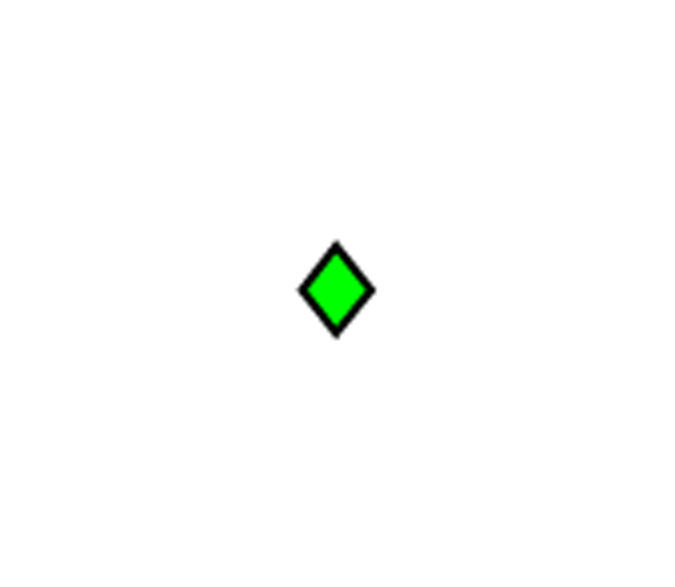
) represents the *division index* of the simulated EIS phase signal displayed in Fig. 4.

The most important results are displayed in Fig. 3. Figure 3a–d show optical and EIS results for one exemplary single *S. pombe* cell over a time course of 120 minutes, whereas Fig. 3e,f show EIS relative-phase recordings of additional cells that experience nuclear division (Fig. 3e) or that do not undergo nuclear division (Fig. 3f). Statistics of nuclear division of all measured cells derived directly from EIS phase signals by using a *division index* (see Methods) are shown in Fig. 3g.

Figure 3a shows time-lapse confocal micrographs before and after nuclear division of an exemplary *S. pombe* cell. Cell division occurred for this specific cell between 40 and 60 min. At 80 min, cytokinesis is completed, and two separate cells appear that are, in most cases, tilted with respect to their long axis.

Figure 3b shows the evolution of the relative magnitude and relative phase signals of the same *S. pombe* cell recorded during 120 minutes over the whole frequency range of 10 kHz to 10 MHz. We observed maximum EIS signal variations at different frequencies, e.g., at 1 MHz for the magnitude and above 5 MHz for the phase signal. We then selected these frequencies of maximal variations for correlation of the EIS measurements with corresponding cellular activities (Fig. 3c).

Figure 3c shows relative impedance phase and magnitude values of the same single cells versus time. Looking first at 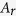 at 1 MHz, the signal descends linearly during a time period of 40 minutes, which is indicative of the elongation of the growing cell in the G2 phase. Then, around 40 min, the growth rate of the cell is decreasing, which is reflected by a slower descent of 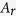 and indicates the entry into the mitotic phase of the cell cycle (green-shaded area). Concurrently, 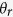 at 5 MHz starts to decrease at 40 min after a preceding continuous increase and reaching a maximum value, and continues to decrease until around 60 min.

To better illustrate the variation in the EIS signals, a plot of 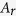 at 1 MHz versus 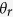 at 5 MHz is shown in Fig. 3d. During the first 40 minutes, 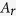 is approximately linearly correlated with 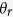, which is indicative of a growth process, i.e., an increase in cell length and volume. Such linear correlation has also been observed for stacking intrinsically invariant beads as shown before. At around 45 min, a 1^st^ turning point in the graph occurs, followed by 2^nd^ turning point at around 60 min. Comparing Fig. 3d to the time course of 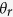 at 5 MHz in Fig. 3c, the two turning points correspond to maximum and minimum values reached at the respective times. The maximum value at around 45 min and the minimum at around 60 min can be assigned to nuclear division processes.

Then, after 60 min and the 2^nd^ turning point in Fig. 3d, the relative magnitude signal, 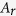, at 1 MHz further decreases with a similar slope as during the initial cell growth before division. The EIS signals indicate that the two separated daughter cells, which were formed through division and are stacked on top of each other, now start to grow again in length.

After approximately 45 minutes, the plots in Fig. 3c–e show features that are different from those of typical linear EIS growth curves (Fig. 2c) or EIS curves that have been obtained upon stacking beads (Supplementary Fig. S3). There is a change in correlation between magnitude and phase signals during cell division, which coincides with a stop in growth and the occurrence of nuclear division processes. Changes in capacitive contributions may result from alterations in the nucleus/cell membranes and nuclear elongation and division. The occurrence of nuclear division and subsequent cytokinesis has been confirmed in the confocal micrographs. More comprehensive data sets of the *S. pombe* growth-division EIS recordings in Fig. 3e are shown in Supplementary Figs S8, S9.

Another feature can be seen in the EIS signal curves in Fig. 3c: The relative magnitude or phase signal shows a drastic change at 72 min, while two separated and tilted daughter cells have been observed optically at 80 min. The drastic signal change can, therefore, be attributed to tilting of the two daughter cells after cell separation, which then no more assume vertical positions in the trap. As the two daughter cells tilt away from the vertical positions in the trap, the relative magnitude increases drastically (the impedance actually drops) as a result of a larger fraction of the orifice becoming permeable to the electric current.

Figure 3e,f show EIS relative-phase recordings of four additional cells that experience nuclear division (Fig. 3e) or two other cells that do not undergo nuclear division (Fig. 3f). Tilting events were observed in many EIS and optical recordings of successful cell division (left two panels of Fig. 3e, Supplementary Fig. S8). However, the cells may also retain their position after cell division and stay immobilized on top of each other without any tilting (right two panels of Fig. 3e; the comprehensive data sets are included in Supplementary Fig. S9). In all cases of cell division, the maximum, which occurs first, and the subsequent minimum were 17.5 ± 5 minutes apart, which is a typical time for mitosis^44^. After cell division, it depended on whether position changes of the daughter cells occurred or not if one could see a large sudden EIS signal change.

The EIS recordings look different, when no cell division occurred (Fig. 3f, Supplementary Figs S10, S11): they do not feature maxima and minima at the expected times in the phase curves, and they do not exhibit the turning points in the plots of 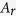 versus 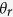 that have been shown in Fig. 3d. Instead, the EIS data show only little variation in later stages of evolution, and tend to form clusters in the 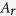-versus-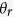 plots (Supplementary Figs S10d, S11d). The corresponding confocal micrographs do not evidence any changes in the nuclei of the corresponding cells (Supplementary Figs S10a, S11a).

We were also able to introduce a quantitative measure to determine directly from the EIS recordings, whether a cell underwent nuclear division or not: We computed the “*division index*” (see Methods section on detection of nuclear division in EIS data) from the relative phase signals at 5 MHz. The *division index* was significantly increased for cells undergoing nuclear division, as compared to those, which did not (Fig. 3g). The respective EIS findings could, in all instances, be confirmed through concurrently recorded confocal images.

### Finite-element modeling of EIS signals

**Figure 4:**
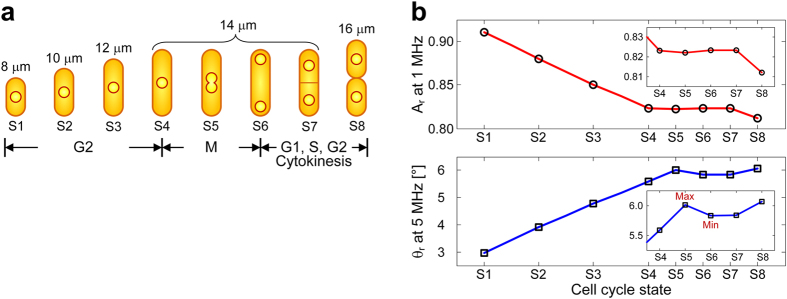
Simulated EIS signals during a cell cycle of *S. pombe*. (**a**) Schematic illustration of the simulated cell-cycle states (S1−S8) corresponding to Fig. 1a. From S1 to S4, the cell grows in the G2 phase from 8 μm to 14 μm length. After S4, the cell enters into the M phase with nuclear elongation. At S5, the nucleus reaches the longest vertical extension before separation. At S6, two daughter nuclei are relocated to the ends of the cell. At S7, a septum appears and separates the daughter cells. At S8, cytokinesis is completed, and two daughter cells are formed and will further grow. (**b**) Simulated cell development curve displaying 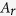 at 1 MHz and 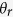 at 5 MHz versus time or cell cycle state. The final tilting of the two daughter cells has not been included in the simulation.

In order to better understand and interpret the EIS and confocal image data, to plausibilize the correlation of the EIS data to cellular processes, such as growth and nuclear division, and to demonstrate the usefulness of the introduced *division index*, we established a finite-element model to simulate the EIS signals at the important cell-cycle states of *S. pombe* (Fig. 4a). The modeled states include cell growth (S1−S4), elongated nucleus (S5), segregated nuclei (S6), cell with septum (S7) and separated daughter cells (S8). The two extracted curves in Fig. 4b are in accordance with Fig. 3c and show similar characteristics: First, one can see a linear decrease of the relative magnitude, 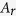, at 1 MHz when the cell length extends from 8 μm to 14 μm (S1−S4). Then in the M phase, the relative magnitude varies only marginally, because the cell remains at a length of 14 μm during nuclear division. In contrast, the relative phase, 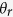, at 5 MHz shows different characteristics during the same time course. The curve reaches a peak when the nucleus is elongated but not yet separated (S5). Afterwards, the curve slightly descends, as the segregated two daughter nuclei move to the ends (S6), and then stays more or less constant during nuclear migration and septum formation (S7). Finally, after cytokinesis (S8), we observe signal characteristics similar to those during the initial cell growth phase (descent of 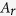 and concurrent ascent of 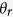) as a consequence of the growth and elongation of the two daughter cells. We also calculated, according to the procedure described in the Methods section, a “theoretical” *division index* value from the simulated relative phase signals obtained through modeling. This value is represented by the green diamond in Fig. 3g and corresponds well to the range of *division index* values that have been obtained from experimental EIS data of cells that underwent nuclear division.

The simulation results obtained by setting up a comparably simple impedance model of a dividing cell (details on the model in the Methods section) are in good agreement with the experimental results: the modeled curve shape and features of 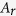 at 1 MHz and 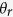 at 5 MHz versus time or cell cycle state in Fig. 4b match up well with the experimentally obtained curves in Fig. 3c and the *division index* value obtained from the model of a dividing cell matches well with experimentally obtained *division indices* of dividing cells. Therefore, the model can be used to plausibilize and explain the obtained EIS data and help to correlate measured impedance values to events in the life cycle of single *S. pombe* cells. The coincidence of experimental data and model proves that the EIS system can detect reliably cell growth and nuclear division.

## Discussion

The single-cell EIS device has been designed for application with *S. pombe* cells and works properly. The narrow bottleneck-like orifices, serving as cell traps, allow for reliable immobilization of *S. pombe* cells in a stand-up mode by hydrodynamic forces. *S. pombe* is not deformed while being trapped, nor is cell growth attenuated in comparison to standard liquid cultures. The elongated shape renders *S. pombe* an ideal model system in comparison, to, e.g., previously used spherical *S. cerevisiae* cells that were found to be prone to rotations and position changes during growth and recording, which largely influenced the impedance signals^13^.

The coplanar microelectrodes lead to a horizontally-distributed electric field, which is orthogonal to the vertically-immobilized cells. *S. pombe* grows exclusively by elongation, and its nucleus divides in three stages: nuclear elongation, nuclear separation and nuclear migration. These changes all occur orthogonally to the electric field. Thus, the chosen configuration of cell immobilization and microelectrodes is ideal for applying wide-band multi-frequency EIS to immobilized cells. Structural modifications of the device may be required for future applications, e.g., with mammalian cells that can be easily deformed during cell-immobilization, which may affect their proliferation.

We studied variations of EIS magnitude and phase signals that have been recorded during cell growth and division. We observed a linear correlation between relative magnitude and phase signals during cell growth, e.g., in the G2 phase, when the cell elongates and its plasma membrane area increases accordingly. After the cell enters the mitotic phase, magnitude and phase signal are no more linearly correlated (Fig. 3d). During nuclear division and cytokinesis, cell growth slows down, and large structural rearrangements happen inside the cell. A local maximum, followed by a signal decrease and minimum appear in the EIS phase curve in Fig. 3c, which then produce two reproducible turning points in the magnitude-versus-phase plot in Fig. 3d (turn to the left and then back to the right) or in Supplementary Figs S8, S9. We observed these two local extrema in all EIS recordings, in which the cells underwent cell division. The average time distance between local maximum and minimum in all these recordings was 17.5 ± 5 minutes, which compares well to the established time of nuclear division of around 17 minutes^44^. Another feature included the drastic signal change upon tilting of the split daughter cells after the end of the cell division process. This drastic change, however, did not happen in all cases of cells that did divide.

The EIS data of all cells that did not show any intracellular or nucleus activity after a growth or elongation period showed almost no variation after around 60 min (Supplementary Figs S10, S11). The phase and magnitude signals of those cells remained approximately at the level reached at the end of the linear growth phase. In all cases the failure to enter into mitosis could be confirmed through the accompanying confocal micrographs. The halt of cell growth in these cases may be due to a failure to pass the G2-M checkpoint^38^,^45^,^46^, at which the entry into the mitotic phase is determined.

The state of cell division, S5, which is shown in the finite-element model in Fig. 4 represents a cell state with an elongated nucleus before it splits into two individual nuclei. This state can be assigned to the local maximum in the simulated EIS growth curve (relative phase signal) (Fig. 4b). Such a local maximum appears in recordings of EIS phase curves (Fig. 3c,e) when the respective cell enters into the mitotic phase and before completion of nuclear division. The local minimum in the simulated phase curve appears before growth of the now separated daughter cells is resumed. The presence of the local maximum and minimum in the phase signal can be used to detect nuclear division directly in the EIS data. To this end, we introduced a “*division index*”, which proved to be significantly higher for cells undergoing nuclear division than for those, which stopped growing (Fig. 3g).

In conclusion, we have presented and experimentally verified a microfluidic device for single-cell immobilization, EIS, and microscopy. This device enables real-time monitoring of immobilized single *S. pombe* cells by using EIS. The measurement system features a spatial resolution of 0.25 μm for measuring the sample height, which corresponds to approximately 5-min growth intervals that can be resolved. The wide-band multi-frequency EIS reliably detects nuclear division and will likely detect other things. The experimental results (EIS and confocal images) are in good agreement with finite-element modeling and simulations, so that simulations and a fairly simple model can be used to correlate obtained EIS data with cell cycle states.

## Methods

### Microfluidic device

The microfluidic device shown in Supplementary Fig. S1a−c was fabricated by using a Glass-Pt-SiN_x_-SU-8-PDMS multi-layer process as shown in Supplementary Fig. S1d. First, 200 nm Pt with a 20-nm-thick TiW adhesion layer underneath were deposited on a 500-μm-thick 4-inch Pyrex wafer and patterned with a lift-off metallization process. Then, a 500-nm-thick SiN_x_ passivation layer was deposited on the whole wafer via plasma-enhanced chemical vapor deposition (PECVD). This layer insulated all metal lines, but was reopened at the sensing regions via reactive-ion etching (RIE) in order to produce defined microelectrodes in the microchannels and contact pads at the chip border. The other metal regions remained insulated, thereby reducing electrical crosstalk and effects from the electrical double layer.

Afterwards, the microfluidic structures (both channels and traps) with a height of ~28 μm were fabricated in SU-8 3025 (MicroChem Co., USA) directly on top of the SiN_x_ layer. Using a mask aligner, the SU-8 patterns were precisely aligned to the Pt microelectrodes on the substrate, which ensured accurate positioning of the bottleneck-like traps (orifice width: 2.5−3 μm) between the stimulus and recording microelectrodes. The wafer was then diced into single chips and bonded to an unstructured polydimethylsiloxane (PDMS) (10:1 w/w, Sylgard^®^ 184, Dow Corning, USA) cover, which sealed the microfluidic channels and completed the chip fabrication. For an irreversible bond, the SU-8 surface of each chip was modified with 3-aminopropyltriethoxysilane (APTES) (Sigma-Aldrich, USA), and the unstructured PDMS layer, comprising punched fluidic inlets and outlets, was activated by oxygen plasma. It is important to notice that no precise alignment is required for the final channel sealing, so that it can be performed under a conventional stereomicroscope. The materials that were used – glass, SU-8 and PDMS – have excellent light transmittance, making the microfluidic system readily accessible to wide field and confocal microscopy.

### Setup

The setup is schematically illustrated in Supplementary Fig. S12. A bonded microfluidic device was first placed on a custom-made Al holder, which fits onto the inverted microscope stages (Olympus IX81 Inverted Microscope, Olympus Corp., Japan, and Nikon A1R + Confocal Microscope, Nikon Corp., Japan) for imaging. Then, the microfluidic device was clamped tightly between the Al holder and a polymethylmethacrylate (PMMA) cover by using screws. On top, a printed circuit board (PCB) comprising manual switches and spring contact probes was positioned. These spring probes contacted the electrode pads on the device when screwed to the Al holder. Via the PCB, an impedance spectroscope (HF2IS, Zurich Instruments AG, Switzerland) and a current amplifier (HF2TA, Zurich Instruments AG, Switzerland) were connected to the microelectrodes on the device. For fluidic access, polytetrafluoroethylene (PTFE) tubing (Bohlender GmbH, Germany) was connected through holes in the PMMA cover to the inlets and outlets of the microdevice.

Media, bead samples or cell suspensions were initially loaded into glass syringes (ILS Microsyringes AG, Germany) and then delivered to the cell-culturing channel at controllable continuous flow, provided by dedicated syringe pumps (neMESYS, Cetoni GmbH, Germany). An important point to mention is that the sample suspension was introduced through the lower-left inlet, while the medium was supplied to the upper-left inlet. In the laminar-flow regime, the upper half of the cell-culturing channel, closer to the traps, was, therefore, perfused with pure medium. The low-pressure conditions for cell capturing were applied via the outlet of the suction channel using a pressure controller (DPI 520, Druck Ltd., UK), supplied with in-house compressed air and vacuum. The pressure controller features high-speed stabilization (10^4^ Pa/s) and high precision (1 Pa) to ensure precise modulation of flow profiles. The instruments, including the impedance spectroscope, syringe pump and pressure controller, were controlled with a PC running custom software.

During the experiment with cells, the whole microscope, as well as the filled syringes affixed on syringe pumps, were placed in an environmental box (Life Imaging Services GmbH, Switzerland) controlling the temperature of 32 °C or 25 °C, respectively.

### Bead and cell trapping

As mentioned before, the fluidic connections serve to guide bead or cell suspensions along the lower half of the cell-culturing channel, at a certain distance from the traps. In order to drag beads and cells towards the trapping sites and to capture one, a low pressure (around −2000 Pa; negative and positive pressures are relative to atmospheric pressure if not specified) was exerted at the suction channel (Supplementary Fig. S2a). Once a bead or cell was captured, the pressure was elevated in a second step to an optimized level (around 500 Pa, which was still lower than that in the cell-culturing channel). At this pressure, the immobilized bead or cell can be retained reliably, and no further bead or cell will be dragged to the trap and captured (Supplementary Fig. S2b). In order to capture multiple beads stacked above each other, an even lower pressure (<−2000 Pa) was applied to the suction channel. The number of stacked beads can be controlled by adjusting the value and application time of the low pressure. The exact values of the required pressure to capture a cell and to reliably retain a cell had to be optimized at the beginning of each set of experiments.

After single-point or long-term measurements, the immobilized bead(s) or cell were released by a slight raise of the applied pressure in the suction channel, which allowed the bead(s) or cell to be dragged away from the trap.

Due to a pressure difference across the various traps^42^, the first trap, producing the highest pressure difference, was usually used for most of the experiments. The calibration of the cell length was done at each used trap, since the traps might have slight differences in terms of geometry and electrical properties of the microelectrodes as a result of process variations during microfabrication.

### Electrical impedance spectroscopy

After immobilization of a single bead or stack of beads or a single cell, an impedance measurement was executed: An alternating current (AC) signal, 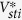 (amplitude 1 V and phase 0), swept over a frequency range from 10 kHz to 10 MHz including 92 sampling frequencies, was applied to the stimulus microelectrode by the impedance spectroscope. The induced signal, which was received by the recording microelectrode situated at the respective trap, was amplified and converted to a voltage signal through the current amplifier, and ultimately recorded by the impedance spectroscope. Therefore, the measured impedance 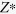 between the stimulus and recording microelectrodes can be expressed as 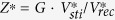, where 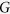 is the gain of the transimpedance amplifier, and 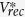 is the recorded voltage signal. This signal is displayed in terms of magnitude, 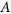, and phase, 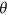, at each applied frequency of the impedance spectroscope. Since the impedance is inversely proportional to the recorded signal 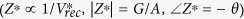, the values of magnitude and phase were directly used as characteristic signals in the measurements.

Impedance measurements over the whole frequency range were performed once for each immobilized sample in characterization experiments. For cell-growth and cell-cycle recordings of *S. pombe* cells, the frequency sweep was carried out every 30 seconds.

EIS data have either been used as raw absolute signals, or as relative signals, calculated with respect to recorded signals from an empty trap. Each measurement of the empty trap was performed right after single-point characterization of each bead or cell. For long-term monitoring of cell growth and cell cycle, the empty trap was measured before and after the recording, and the respective values were used as a reference. The impedance signal of the empty-trap measurement is displayed as magnitude *A*_*e*_ and phase *θ*_*e*_. Thus, the relative magnitude 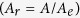 was calculated by dividing the signal magnitude when a sample was trapped, by the signal magnitude of the empty trap. The relative phase 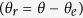 was calculated by subtracting the phase signal of the empty trap from that of the trap with an immobilized sample. Obvious signal jumps or steps, which could be clearly assigned to additional capturing of small particles or cell debris during EIS recording, were removed manually.

### Equivalent circuit model

An ECM has been used to better understand variations in the EIS signals of an immobilized *S. pombe* cell (Fig. 1e). The measured impedance between stimulus and recording microelectrodes is depending on the nature of the microelectrodes themselves 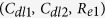, on that of the cell 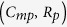, and on that of the trap 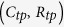. The contribution of the cells to the signal has been determined by comparing signals from trapped cells to those of the empty trap. Signals obtained from empty traps have been utilized as reference values for all measurements.

### Imaging

For the characterization experiment with beads, a CCD camera (F-View II, Soft Imaging System GmbH, Germany) on the Olympus IX 81 microscope (LUCPLFLN 60X objective, 0.7 NA, correction ring adjusted to 0.5 mm to match the thickness of device substrate), was used to acquire bright-field images of immobilized beads. For the calibration experiment with *S. pombe* cells, the Nikon A1R + confocal microscope (CFI S Plan Fluor ELWD ADM 40X objective, 0.6 NA, correction ring adjusted to 0.5 mm) was used to scan each immobilized cell (xy-resolution of 0.1 μm, z-stack interval of 1 μm). For cell-growth and cell-cycle recordings of *S. pombe* cells, the same confocal microscope with the same objective was used to scan the immobilized cell every 20 min (64 × 64 pixels, xy-resolution of 0.4 μm, z-stack interval of 0.5 μm).

The length of *S. pombe* cells for calibration and validation of the measurement system has been extracted manually from the z-stacks of the confocal micrographs. The following methods were used to optimize accuracy: (i) The bottom boundary of the cell was defined by a sharp increase of fluorescence intensity and the appearance of a ring-shaped structure that is caused by the cell plasma membrane; (ii) For an undivided cell, the total length of the cell can be defined as two times the distance from the bottom boundary to the fluorescently brightest plane of the nucleus, because the nucleus is maintained in the cell center by cytoplasmic microtubules; (iii) All cells, no matter at which stage of division, are geometrically symmetric along the z-axis; (iv) After division, the upper nucleus is visible due to the enhanced fluorescence of the nuclear membrane, even though the intensity is still lower compared to the lower nucleus.

### Bead preparation

Commercial monodisperse PS beads (Fluka, Sigma-Aldrich Co., Switzerland) with known diameters of 6.084 ± 0.082 μm, 7.177 ± 0.086 μm and 8.020 ± 0.098 μm (calibration values from the manufacturer’s datasheet) were employed for EIS characterization in this device. Before loading the samples into the syringe, beads were suspended in yeast culture medium. Potential bead-clusters in the suspension were mechanically separated for 5 minutes in an ultrasonic bath at room temperature.

### Cell preparation

An *S. pombe* strain (h972, haploid h+) was used in the experiments. The gene *ERG11* had been genetically modified by fusing its C-terminus with the GFP gene. The resulting protein Erg11-GFP inserts into the endoplasmic reticulum (ER) so that the boundaries of the nucleus and the cell became visible through green fluorescent protein. In particular, the nuclear membrane (which is part of the ER) is clearly visible in this strain. Cell suspension was grown in a medium made of 0.5% w/v Yeast Extract (YE) (Bacto^TM^, BD, USA), 2% glucose (Sigma-Aldrich Co., Germany), 150 mM NaCl solution (Sigma-Aldrich Co., Germany) and 0.05% w/v Pluronic^®^ F127 (Sigma-Aldrich Co., Germany) at 32 °C and at 25 °C. Pluronic^®^ F127 solution was used to prevent hydrophobic components in the medium from sticking together. We have validated that the F127 solution at the given concentration had no impact on cell growth of *S. pombe*.

### Modeling and simulation

Fluid dynamics in the microfluidic device during capturing and retaining of a cell have been modeled using 2D CFD simulation in COMSOL Multiphysics software (COMSOL, Inc., USA). “Incompressible Navier‒Stokes” physics from the MEMS Module was applied in simulation. To show the basic working principle, the simulated geometry was simplified to the section of interest containing only cell-culturing and suction channels with three traps (Supplementary Fig. S2). Subdomains were assigned with a density of 10^3^ kg m^−3^ and a dynamic viscosity of 10^−3^ Pa∙s (for water). The no-slip boundary condition was set for the walls of channels and traps. A flow rate of 1 μL min^−1^ was applied at the inlet of the cell-culturing channel in a laminar flow regime. By varying the pressure, applied to the suction channel, we obtained pressure profiles and stream lines referring to the volumetric flow rates. The pressures used in experiments were different from the applied pressures in simulation, due to the longer length of channels, tubing connections and fabrication variations.

Based on the cell-cycle states during the cell growth and division of *S. pombe* in Fig. 1a, a finite-element model was established in COMSOL to simulate EIS recordings of the *S. pombe* cell cycle (Fig. 4). The modeled geometry (Supplementary Fig. S13) is analogous to the one schematically shown in Fig. 1e. Cell growth in the G2 phase is mimicked by a continuous increase of the cell length from 8 μm to 14 μm with an interval of 2 μm. The modeled cell keeps a constant diameter of 4.6 μm. For nuclear division, we varied the center-to-center distance between the two nuclei by two values (2 μm and 8 μm), while keeping the diameter of the nuclei constant along all simulated states. A membrane in the middle of the cell was added to mimic the appearance of the septum before cytokinesis. In the final state, the model consisted of two stacked cells with a length of 8 μm for each. EIS signals were simulated at 19 frequencies in the range of 10 kHz to 10 MHz. The double-layer capacitance of the microelectrodes and the parasitic components of the used measurement system were extracted by fitting the measurement results of the empty trap to the ECM using MATLAB software (MathWorks, Inc., USA). The critical parameters used in the model are presented in Supplementary Note S2 and Supplementary Table S3.

### Detection of nuclear division in EIS data

To detect the occurrence of nuclear division in the EIS data, we devised a simple heuristic method, which was motivated by our simulations (see Fig. 4). We observed that the derivative of the phase signal at 5 MHz changed sign between the S4 and S6 cell cycle states, resulting in a local maximum in the phase signal. We quantified the presence of the local extrema by the “*division index*”: First, the relative phase at 5 MHz was smoothed by using a Savitzky-Golay filter of order 2 with a span of 40 (20 min) to remove small local extrema introduced by high frequency noise. The resulting signal was then normalized by using its global minimum and maximum to the range of [−1 0]. We then detected local maxima and, in case that the temporal smoothing did not remove all fluctuations introduced by noise, we removed those that were temporally too close (10 data points or 5 min) to larger local maxima, or too small with respect to neighboring values (they had to be larger by at least 10^−4^). The same procedure was repeated to detect local minima. The *division index* was then defined as the difference between the first local maximum and its directly succeeding local minimum. If no local minimum was detected, or if the first local minimum after the first local maximum was located at the last sample of the recording, the *division index* was set to 0. A local minimum location at the last recording sample was observed in most recordings that did not feature nuclear division, as, in these cases, the resulting phase signal stayed nearly constant after cell growth had stopped.

### Statistical analysis

All error bars are standard deviations. “
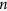
” refers to the number of samples if not specified otherwise. Significance criteria rely on a two-sample Student *t*-test using *p < 0.05, **p < 0.01, ***p < 0.001, and ****p < 0.0001.

## Additional Information

**How to cite this article**: Zhu, Z. *et al.* Time-lapse electrical impedance spectroscopy for monitoring the cell cycle of single immobilized *S. pombe* cells. *Sci. Rep.* **5**, 17180; doi: 10.1038/srep17180 (2015).

## Supplementary Material

Supplementary Information

Supplementary Movie S1

Supplementary Movie S2

Supplementary Movie S3

Supplementary Movie S4

Supplementary Movie S5
