# Supplementary material for: Time-lapse electrical impedance spectroscopy for monitoring the cell cycle of single immobilized S. pombe cells
Source: Sci Rep. 2015 Nov 26;5:17180. doi: 10.1038/srep17180 (PMC4660434; doi:10.1038/srep17180)
Supplement: Supplementary Information [file srep17180-s1.pdf]

Supplementary Material

Time-lapse electrical impedance spectroscopy for monitoring the cell cycle of single immobilized *S. pombe* cells

Zhen Zhu[a], Olivier Frey[a], Niels Haandbaek[a], Felix Franke[a], Fabian Rudolf[b], and Andreas Hierlemann[a]

[a]Bio Engineering Laboratory and [b]Computational Systems Biology Group, Department of Biosystems Science and Engineering, ETH Zurich, Mattenstrasse 26, CH-4058 Basel, Switzerland.

Table of Contents

Supplementary Figure S1: Photograph and schematic of the microfluidic EIS device 3

Supplementary Figure S2: CFD simulation 4

Supplementary Figure S3: EIS performance characterization using beads 5

Supplementary Figure S4: Micrographs of different combinations of two stacked beads 6

Supplementary Figure S5: Raw and relative EIS signals over the whole frequency range of three and four 6-μm stacked beads shown in **Supplementary Fig. S3d** 7

Supplementary Figure S6: Raw and relative EIS signals over the whole frequency range of all samples shown in **Supplementary Fig. S3e** 8

Supplementary Figure S7: Raw and relative EIS signals over the whole frequency range of all samples shown in **Supplementary Fig. S3f** 9

Supplementary Figure S8: Monitoring cell growth and division of a single *S. pombe* cell by using EIS 10

Supplementary Figure S9: Monitoring cell growth and division of a single *S. pombe* cell by using EIS 11

Supplementary Figure S10: Monitoring cell growth of a single *S. pombe* cell by using EIS 12

Supplementary Figure S11: Monitoring cell growth of a single *S. pombe* cell by using EIS 13

Supplementary Figure S12: Schematic of the setup 14

Supplementary Figure S13: Schematic of the finite-element model 15

Supplementary Table S1: Linear regression on EIS measurement data with the heights of single and stacked beads 16

Supplementary Table S2: Measurement data of EIS versus calibration data of the same batch of beads from a Coulter Multisizer II according to the provided data sheet 17

Supplementary Table S3: Parameters used for EIS modeling and simulation of the cell cycle EIS signals displayed in **Fig. 4**.. 18

Supplementary Movie S1: Trapping an *S. pombe* cell vertically... 19

Supplementary Movie S2: 3D animation showing the immobilization, growth and EIS measurement of an *S. pombe* cell 19

Supplementary Movie S3: Trapping 2 × 6 µm beads 19

Supplementary Movie S4: Trapping 4 × 6 µm beads 19

Supplementary Movie S5: Trapping 2 × 6 µm beads together with a particle 19

Supplementary Note S1: Sensitivity of EIS... 20

Supplementary Note S2: Parameters for modeling.. 22

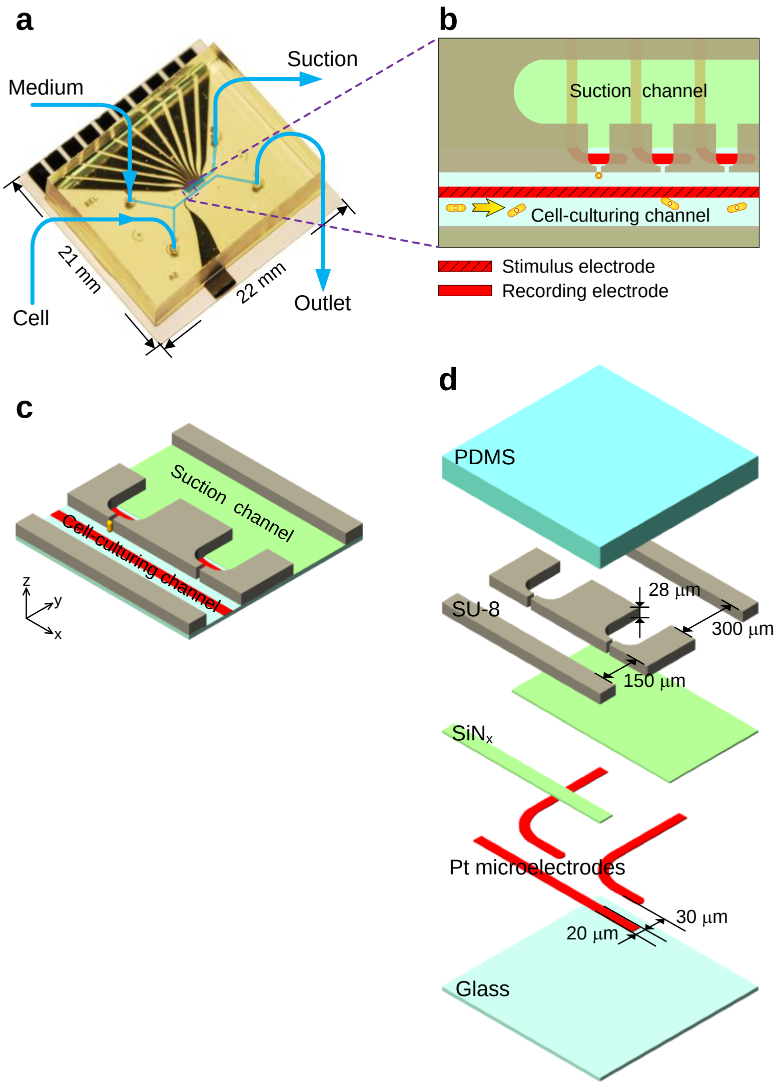

Supplementary Figure S1: Photograph and schematic of the microfluidic EIS device. (a) Photograph of the device with the fluidic connections. (b) Schematic top view of the microfluidic cell-trapping area. A common stimulus microelectrode and individual recording microelectrodes are used for EIS implementation. (c) 3D close-up of an immobilized *S. pombe* cell at the orifice of one trap. The PDMS cover is not present for better visibility. (d) Exploded view of the microdevice illustrating different layers and important dimensions (see Methods for details).

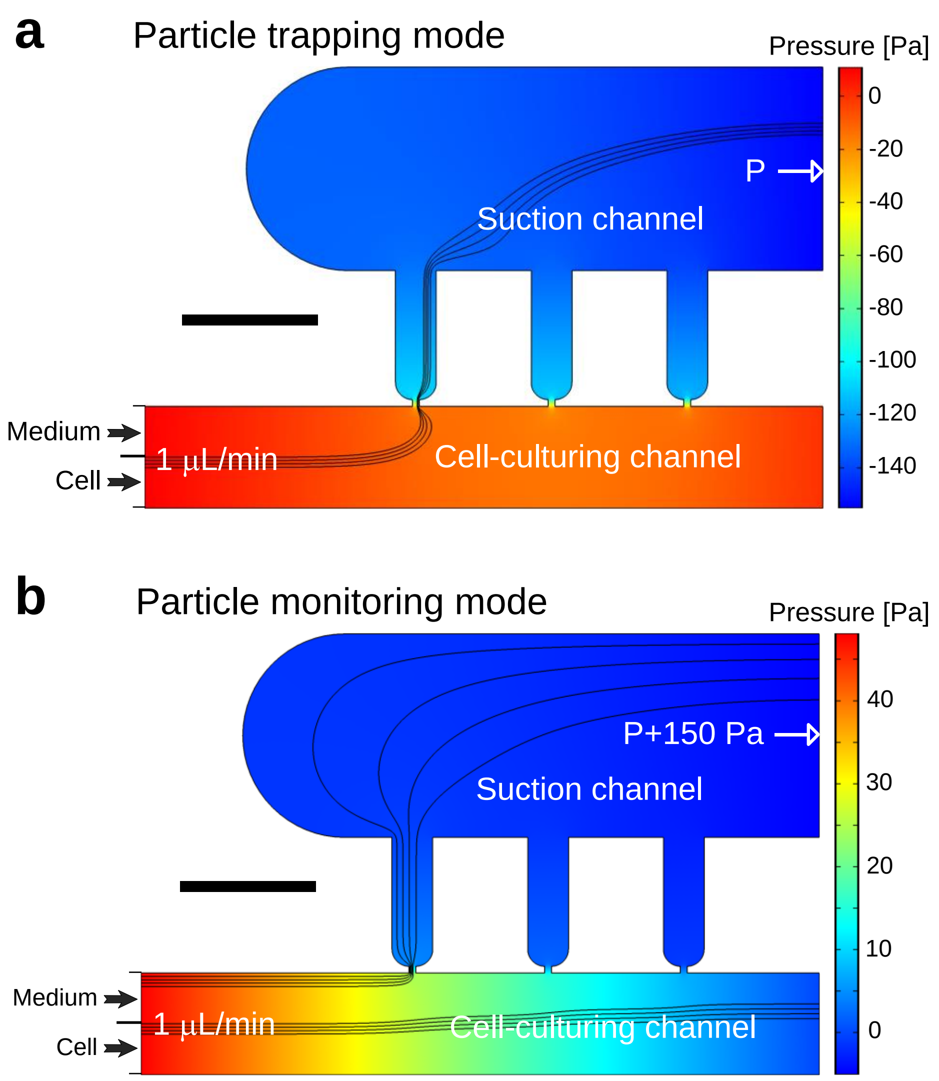

Supplementary Figure S2: CFD simulation in COMSOL software showing the flow streams (black lines) that depend on adjusting the pressure applied to the suction channel during capturing and retaining a single cell. (a) When a lower pressure, ***P***, is applied at the suction channel, cells can be dragged towards the trap and are then captured. (b) Once a cell is immobilized, ***P*** is elevated to an optimized value (herein, ***P***+150 Pa), which ensures that the cell is reliably retained without being dragged away and that no additional cells become trapped at the same site. The optimized pressure is still lower than the pressure in cell-culturing channel to generate a sufficiently high pressure difference across the trap to retain the immobilized cell, but not large enough to deviate the cell stream towards the traps. Scale bar is 200 µm.

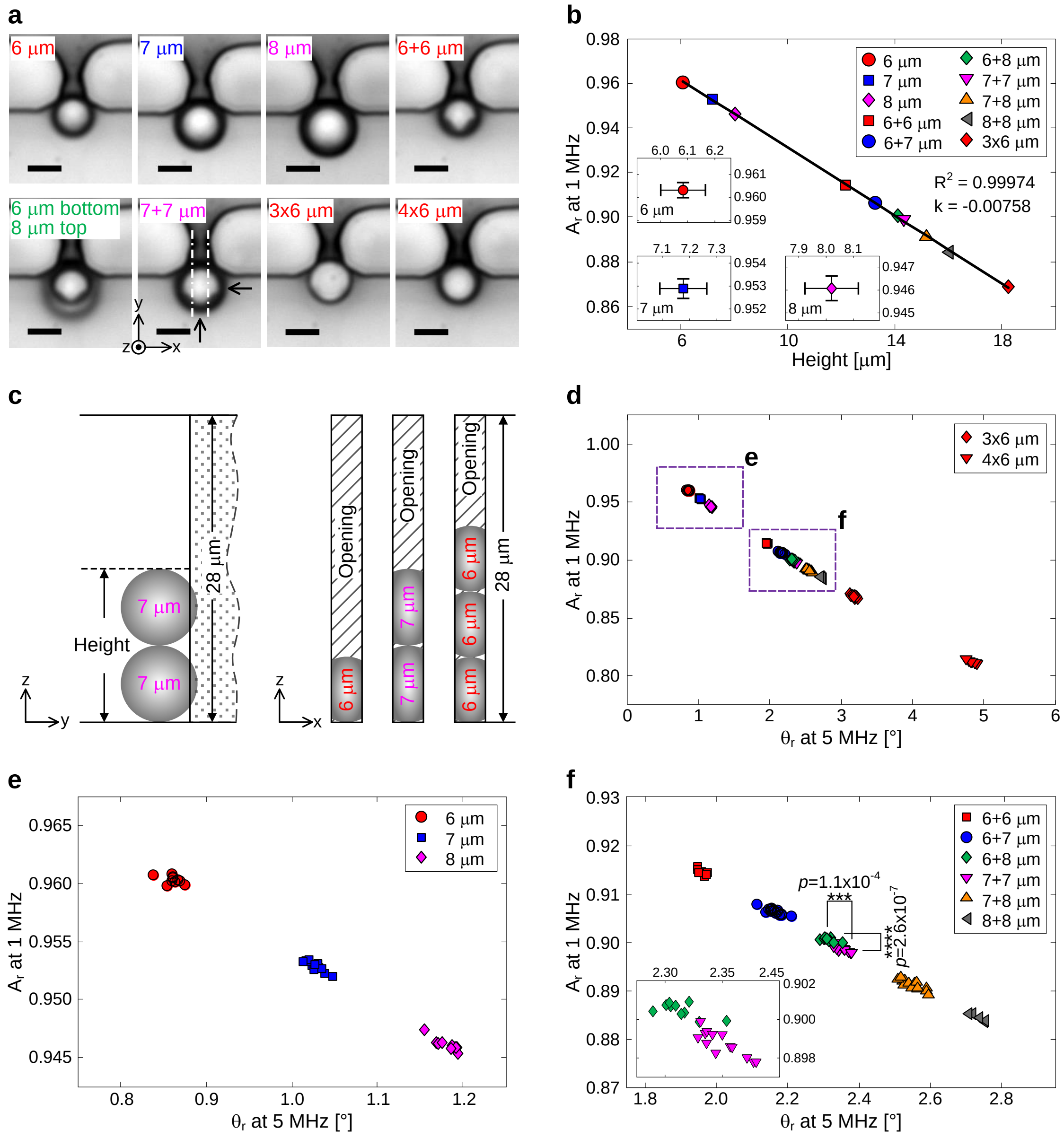

Supplementary Figure S3: EIS performance characterization by measuring immobilized PS beads. (a) Micrographs of immobilized beads with different combinations (imaged from the bottom using an inverted microscope). The other micrographs are shown in **Supplementary Fig. S4**. Scale bar is 5 µm. (b) Linear regression between the measurement results from EIS and the height of single and stacked beads (see **Supplementary Table S1** for details and sample sizes). Inserts show close-ups for 6-, 7- and 8-µm beads. Error bars along x-axis represent the s.d. from the datasheet, while error bars along the y-axis represent the s.d. obtained in EIS measurements. (c) Schematic side and front views of single and stacked beads, illustrating the height of beads and the cross-sectional opening of the trapping orifice. (d) Relative magnitude at 1 MHz versus relative phase at 5 MHz for the classification of all arrangements of measured beads. (e) Close-up views of single immobilized beads with three different diameters: 6 µm, 7 µm, and 8 µm. (f) Close-up views of two stacked beads with different combinations of diameters. Box is further close-up of 6+8 µm and 7+7 µm bead stacks showing that they can be differentiated by using a two-sample *t*-test even with only a slight difference in the obstructed area of the orifice and overall height.

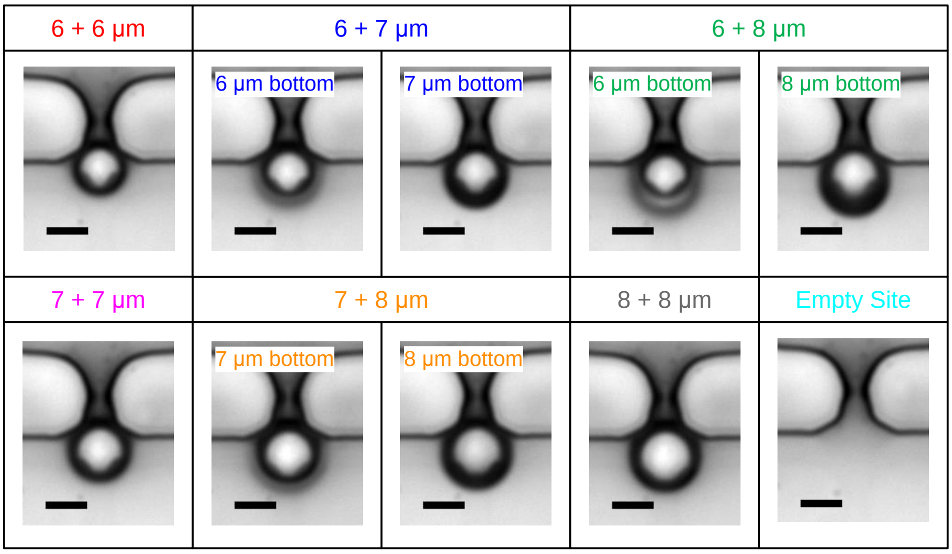

Supplementary Figure S4: Micrographs of different combinations of two stacked beads.

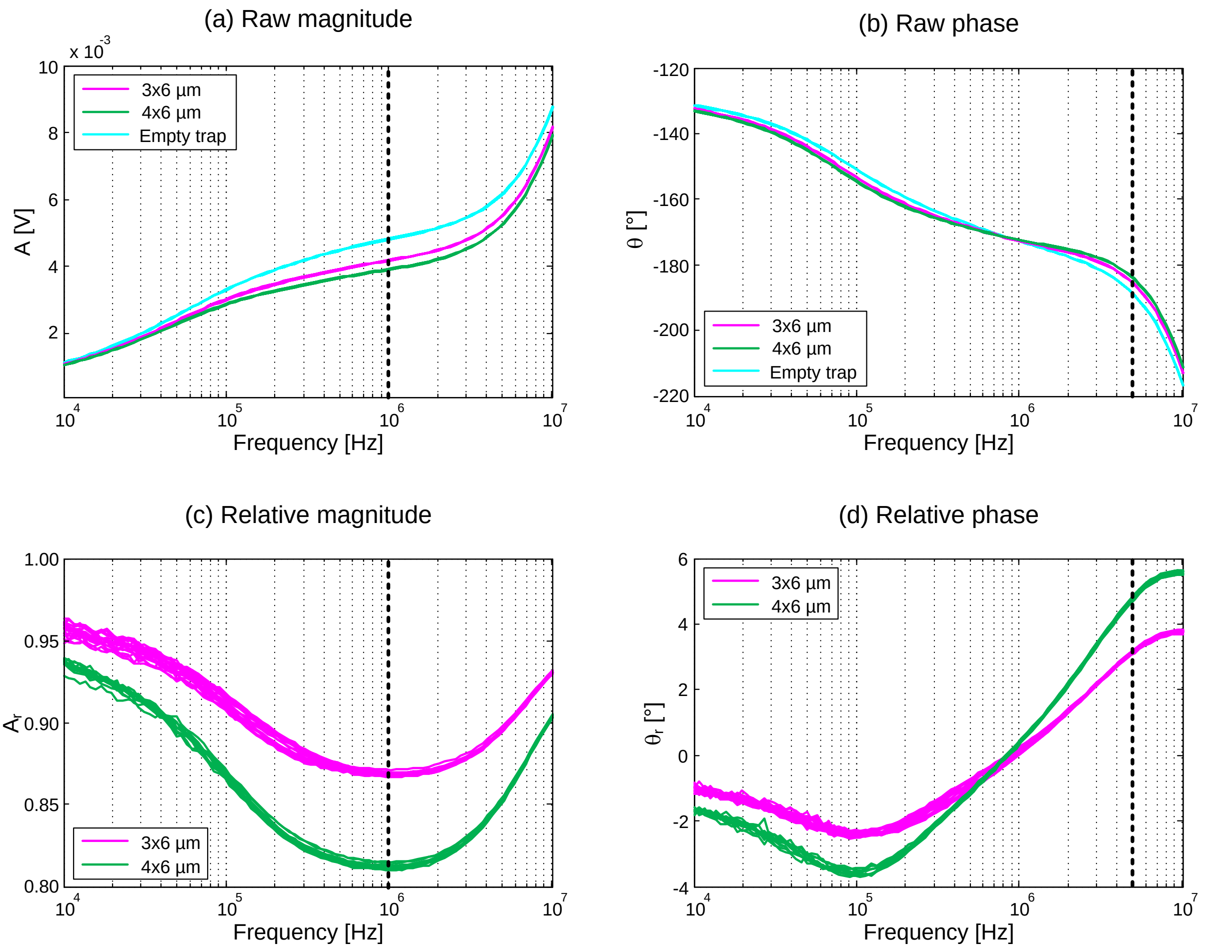

Supplementary Figure S5: Raw and relative EIS signals over the whole frequency range of three and four 6-µm stacked beads shown in **Supplementary Fig. S3d**.

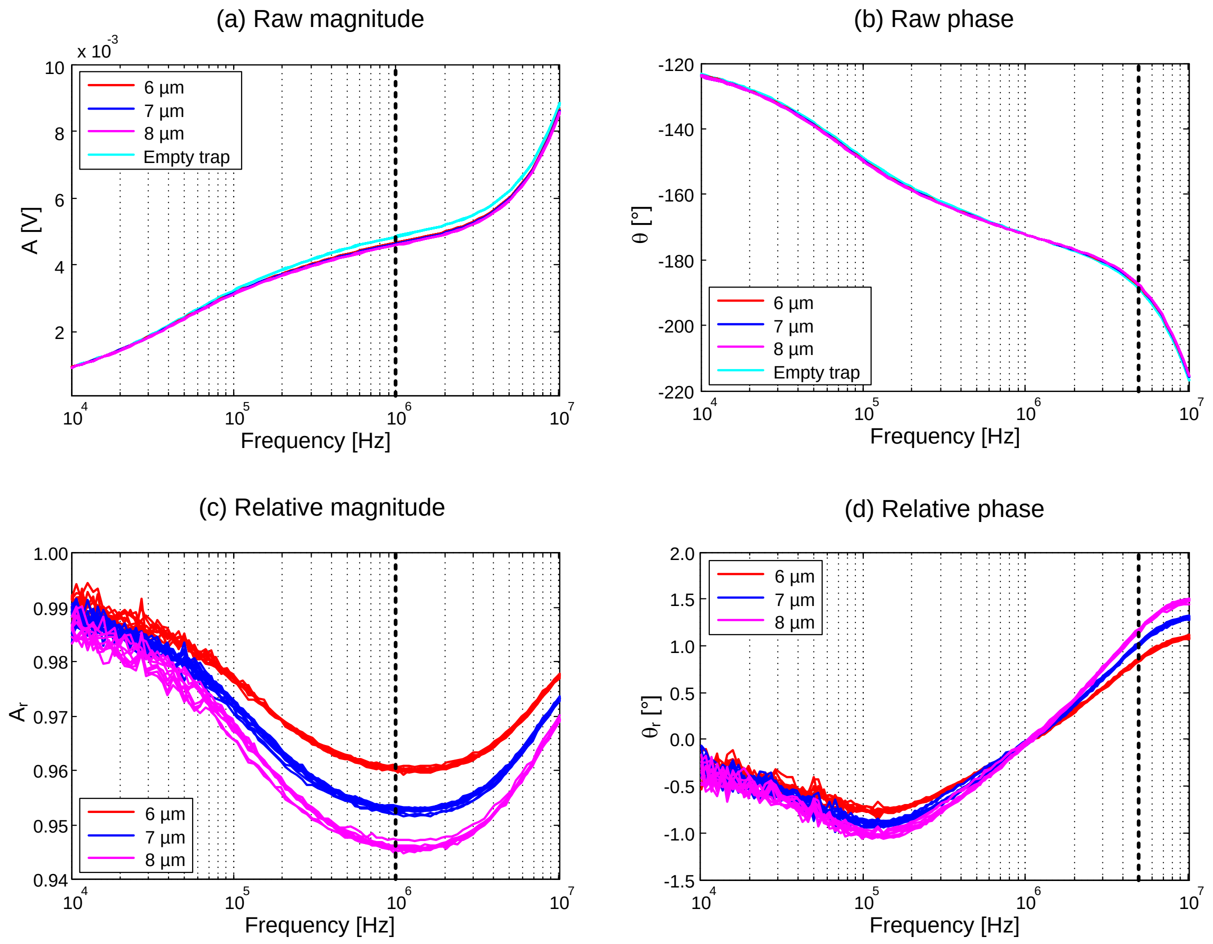

Supplementary Figure S6: Raw and relative EIS signals over the whole frequency range of all samples shown in **Supplementary Fig. S3e**.

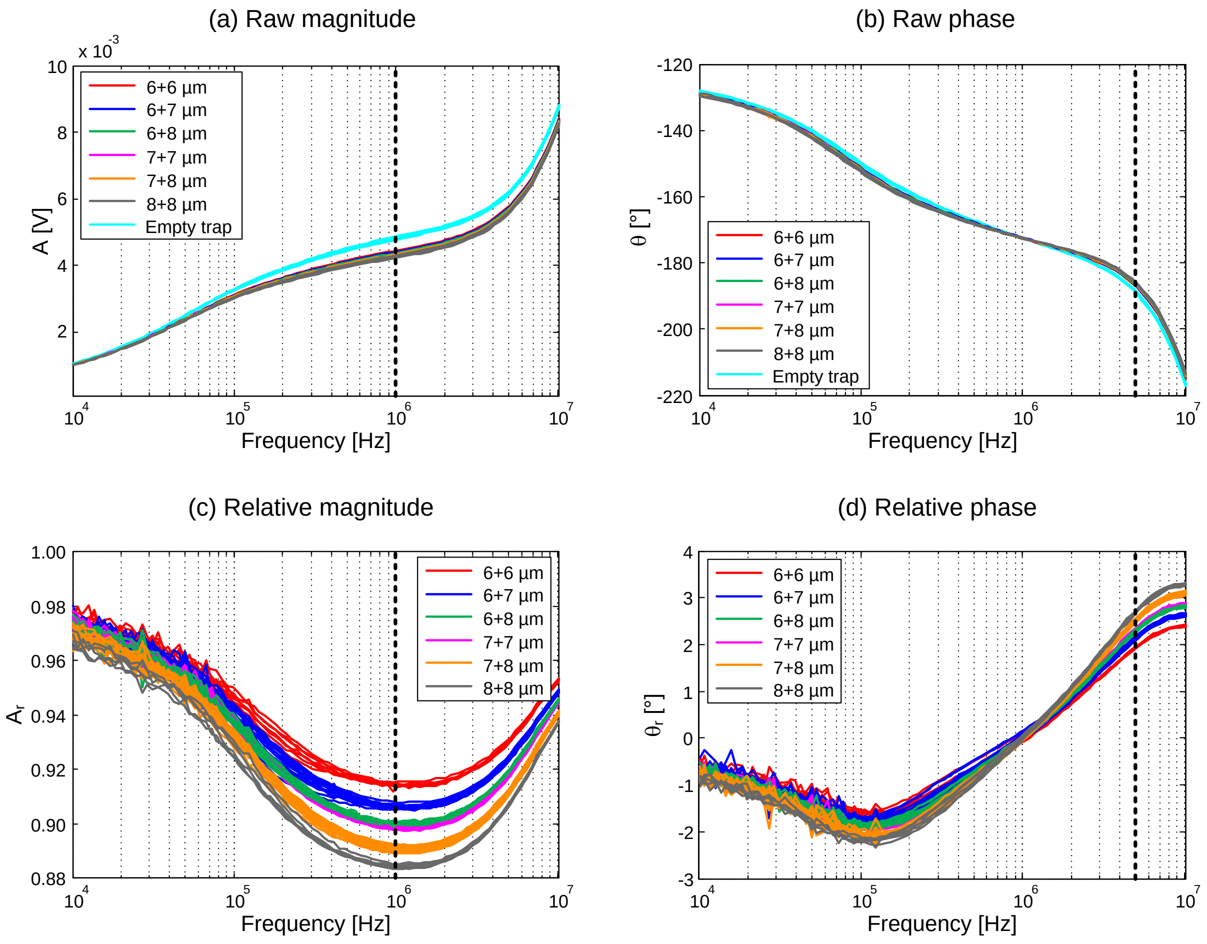

Supplementary Figure S7: Raw and relative EIS signals over the whole frequency range of all samples shown in **Supplementary Fig. S3f**.

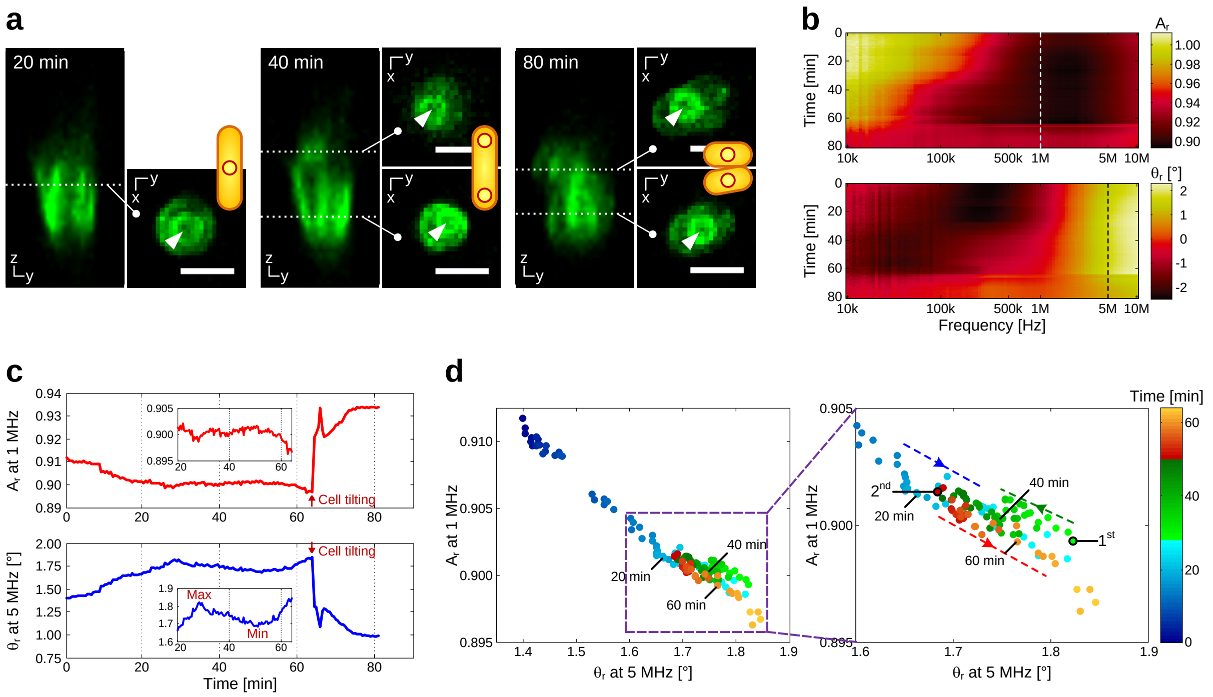

Supplementary Figure S8: Monitoring cell growth and division of a single *S. pombe* cell by using EIS. (a) Time-lapse confocal micrographs and corresponding cell cartoons at 20, 40 and 80 min after trapping of the cell. (b) Recorded EIS signals over the whole frequency range from 10 kHz to 10 MHz, plotted as relative magnitude and relative phase signals with respect to empty-trap reference signals. (c) Growth curve displayed as A_r at 1 MHz and θ_r at 5 MHz versus time, respectively. The inserts highlight the temporal evolution of EIS signals during nuclear and cell division. (d) Scatter plot of A_r at 1 MHz versus θ_r 5 MHz during cell growth, nuclear division and cytokinesis. The close-up view indicates the trends in the correlation and the resulting turning points (1st and 2nd).

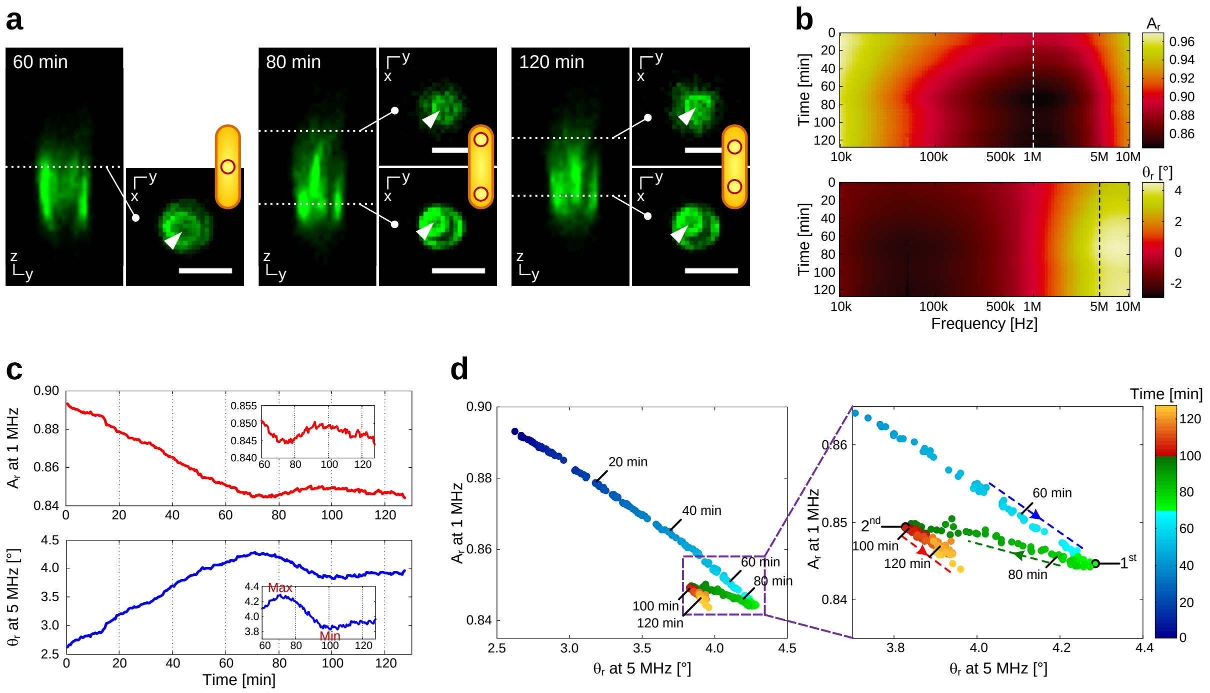

Supplementary Figure S9: Monitoring cell growth and division of a single *S. pombe* cell by using EIS. (a) Time-lapse confocal micrographs and corresponding cell cartoons at 60, 80 and 120 min after trapping of the cell. The two daughter cells did, in this case, not tilt and change position after cytokinesis. (b) Recorded EIS signals over the whole frequency range from 10 kHz to 10 MHz, plotted as relative magnitude and relative phase signals with respect to empty-trap reference signals. (c) Growth curve displayed as A_r at 1 MHz and θ_r at 5 MHz versus time, respectively. The inserts highlight the temporal evolution of EIS signals during nuclear and cell division. (d) Scatter plot of A_r at 1 MHz versus θ_r 5 MHz during cell growth, nuclear division and cytokinesis. The close-up view indicates the trends in the correlation and the resulting turning points (1st and 2nd).

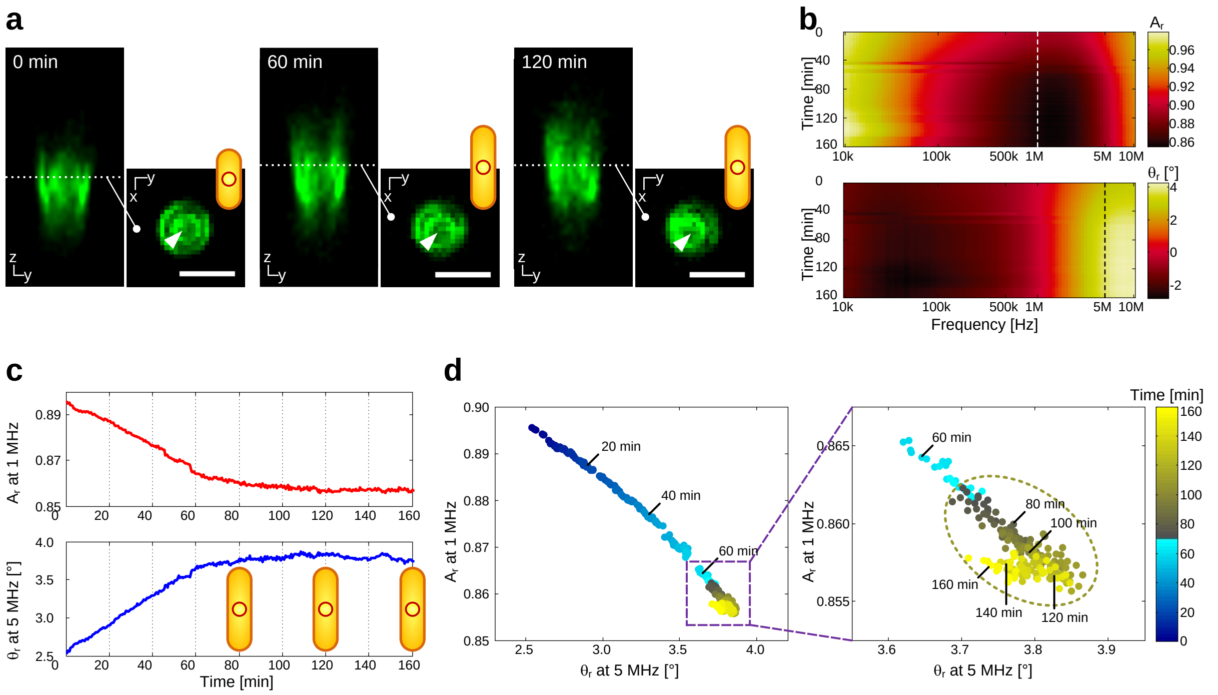

Supplementary Figure S10: Monitoring cell growth of a single *S. pombe* cell by using EIS. It shows a failure of entering the mitotic phase after cell growth in the G2 phase. (a) Time-lapse confocal micrographs and corresponding cell cartoons at 0, 60 and 120 min after trapping of the cell. (b) Recorded EIS signals over the whole frequency range from 10 kHz to 10 MHz, plotted as relative magnitude and relative phase signals with respect to empty-trap reference signals. (c) Growth curve displayed as A_r at 1 MHz and θ_r at 5 MHz versus time, respectively. Cell cartoons show no nuclear activity and are again based on time-lapse confocal micrographs. (d) Scatter plot of A_r at 1 MHz versus θ_r 5 MHz showing the halt of data variation after around 70 min and a data cluster formed subsequently.

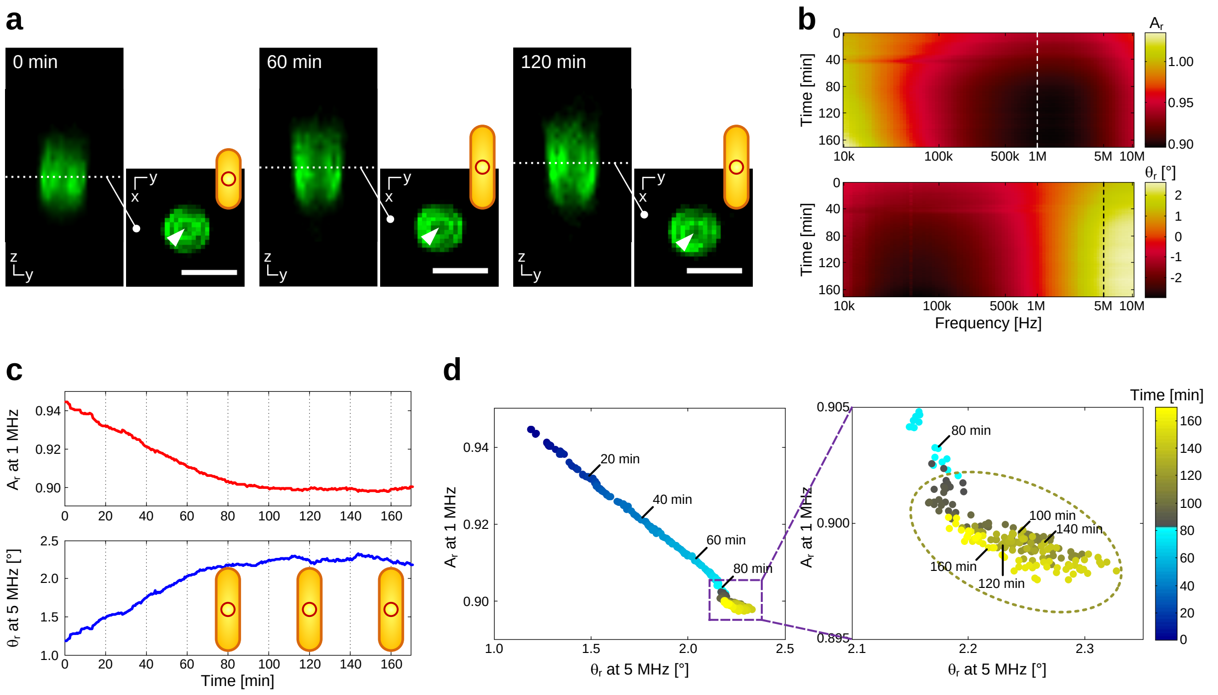

Supplementary Figure S11: Monitoring cell growth of a single *S. pombe* cell by using EIS. It shows a failure of entering the mitotic phase after cell growth in the G2 phase. (a) Time-lapse confocal micrographs and corresponding cell cartoons at 0, 60 and 120 min after trapping of the cell. (b) Recorded EIS signals over the whole frequency range from 10 kHz to 10 MHz, plotted as relative magnitude and relative phase signals with respect to empty-trap reference signals. (c) Growth curve displayed as A_r at 1 MHz and θ_r 5 MHz versus time, respectively. Cell cartoons show no nuclear activity and are again based on time-lapse confocal micrographs. (d) Scatter plot of A_r at 1 MHz versus θ_r 5 MHz showing the halt of data variation after around 80 min and a data cluster formed subsequently.

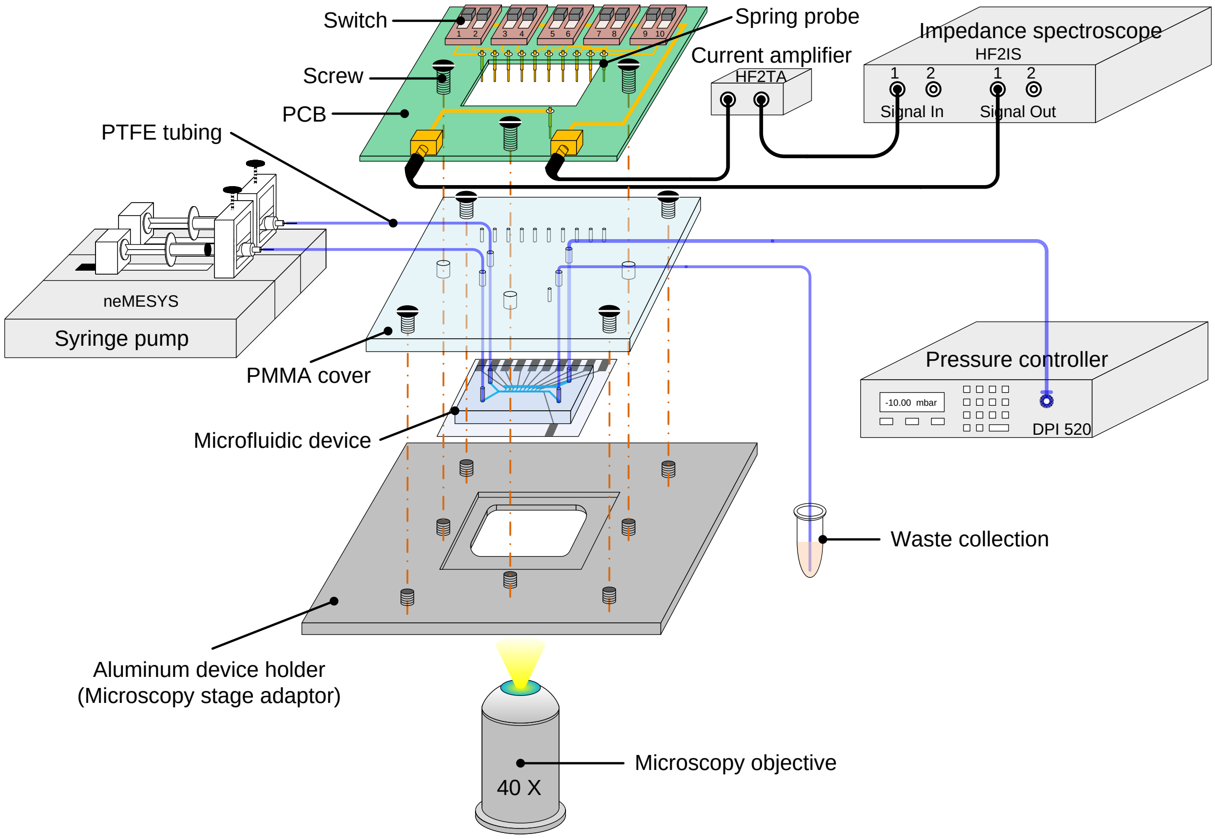

Supplementary Figure S12: Schematic of the setup. The bonded microfluidic device is first placed on a custom-made aluminum holder, which fits onto the inverted microscope stage. Then, the microfluidic device is affixed by screws between the aluminum holder and a PMMA cover. On a PCB, manual switches and spring contact probes are soldered. These spring contact probes interface to the electrode pads on the device when the PCB is screwed down to the aluminum holder. Via the PCB, an impedance spectroscope and a current amplifier are connected to the microelectrodes on the device. PTFE tubing is used to connect inlets and outlets of the device to two corresponding syringes, a pressure controller and a waste container. Sample suspensions are initially loaded into glass syringes and then delivered to the cell-culturing channel by syringe pumps. The outlet of the suction channel is connected to a pressure controller, supplied with in-house compressed air and vacuum. The outlet of the cell-culturing channel is connected to waste. The impedance spectroscope, the pressure controller and the syringe pumps are controlled with a PC running custom software.

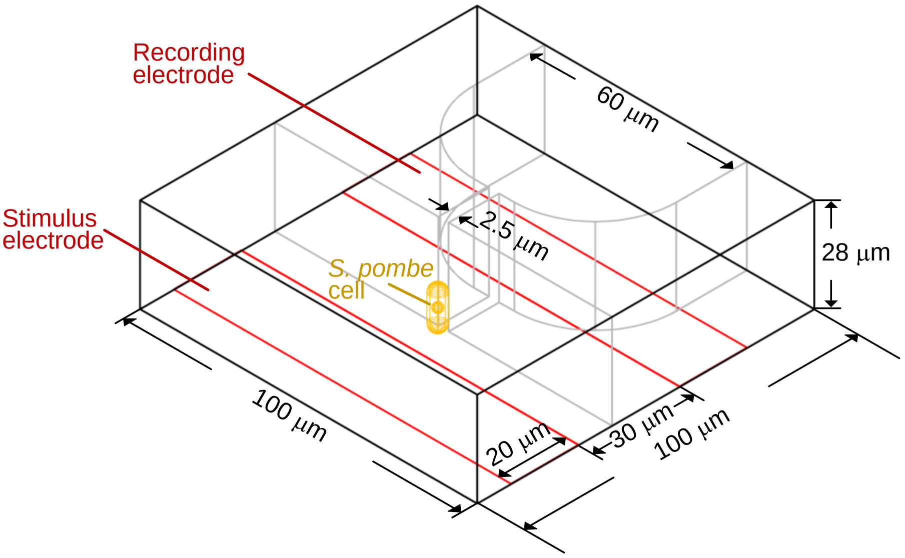

Supplementary Figure S13: Schematic of the finite-element model used for simulation.

Supplementary Table S1: Linear regression on EIS measurement data with the heights of single and stacked beads

Beads	Number of samples, n	Mean A_r at 1 MHz	s.d.	Height [μm]
6 μm	10	0.96032	0.00032943	6.084
7 μm	14	0.95289	0.00042730	7.177
8 μm	10	0.94607	0.00053645	8.02
6+6 μm	7	0.91452	0.00061732	12.168
6+7 μm	18	0.90643	0.00059217	13.261
6+8 μm	10	0.90048	0.00037152	14.104
7+7 μm	13	0.89875	0.00065552	14.354
7+8 μm	15	0.89117	0.00098429	15.197
8+8 μm	5	0.88441	0.00078997	16.040
3×6 μm	20	0.86881	0.00087638	18.252
Coefficient of determination R^2				0.99974
4×6 μm	10	0.81215	0.00162338	24.336
Coefficient of determination R^2				0.99697

Supplementary Table S2: Measurement data of EIS versus calibration data of the same batch of beads from a Coulter Multisizer II according to the provided data sheet

	EIS data: A_r at 1 MHz			Calibration result from datasheet		
Beads	Mean	s.d.	CV [%]	Mean diameter [µm]	s.d. [µm]	CV [%]
6 µm	0.96032	0.00032943	0.034304	6.084	0.082	1.4
7 µm	0.95289	0.00042730	0.044842	7.177	0.086	1.2
8 µm	0.94607	0.00053645	0.056703	8.02	0.098	1.2

Supplementary Table S3: Parameters used for EIS modeling and simulation of the cell cycle EIS signals displayed in **Fig. 4**

	Geometric parameter [μm]	Conductivity σ [S/m]	Relative permittivity ε_r
Cell	Diameter: 4.6		
Cell wall	Thickness: 0.22	0.053	60
Plasma membrane	Thickness: 8×10^{-3}	3×10^{-7}	3
Cytosome		0.3	70
Nucleus	Diameter: 2.6	1.0	70
Nuclear membrane	Thickness: 8×10^{-3}	3×10^{-7}	3
Medium		0.53	80

Supplementary Movie S1: Trapping an *S. pombe* cell vertically

Supplementary Movie S2: 3D animation showing the immobilization, growth and EIS measurement of an *S. pombe* cell

Supplementary Movie S3: Trapping 2×6 µm beads

Supplementary Movie S4: Trapping 4×6 µm beads

Supplementary Movie S5: Trapping 2×6 µm beads together with a particle. Such case is discarded for data analysis.

Supplementary Note S1: Sensitivity of EIS

EIS performance has been characterized with monodisperse PS beads with known diameters of 6.084±0.082 µm, 7.177±0.086 µm, and 8.020±0.098 µm (values from the manufacturer's datasheet). As beads consist of solid plastic material, they do not produce any phase changes in the impedance spectra so that only the relative magnitude A_r was considered. Three sets of experiments with different bead arrangements were performed: (i) single beads with three different diameters, (ii) two beads (three different diameters each) vertically stacked, and (iii) three and four 6-µm beads vertically-stacked to cover the expected cell length range (**Supplementary Figs. S3,S4 & Movie S3−5**).

The relative magnitude A_r at 1 MHz correlates linearly to the height of the single and stacked beads (coefficient of determination, R^2=0.99974) (**Supplementary Fig. S3 & Table S1,S2**). The spatial resolution of our EIS system was determined to be 0.25 µm, calculated from three times the mean standard deviation (s.d.) of measurements of the bead arrangements and the slope of a corresponding linear-curve fit (k) ($-3\times$s.d./k). The tight correlation between relative signal magnitude A_r at 1 MHz and the stack height can be explained by the bottleneck-like geometry of the trap and the arrangement of the microelectrodes. The electrical impedance between the stimulus and recording electrodes at a trap is dominated by the cross-sectional area of the trapping orifice, through which the electric current is constrained to flow. Any variation of the cross-sectional area of the orifice thus will induce a change in the EIS signal. Since an orifice features an invariant width (**Supplementary Fig. S3a**), height increase of an immobilized sample (stacked beads or growing cell) leads to a linear decrease of the cross-sectional opening of the orifice and a concurrent linear variation in the EIS signal (**Supplementary Fig. S3c**). Therefore, impedance measurements in this configuration are highly sensitive to the height or height changes of a sample immobilized at the orifice, and the resulting EIS signal magnitude can be directly correlated to the height of the bead stack or the length of a vertically immobilized cell in later experiments. In the case of four stacked 6-µm beads, we observed slightly nonlinear characteristics of the EIS signal (R^2=0.99697, **Supplementary Table S1**), which can be attributed to the total height of the stacked beads (~24 µm) approaching the overall height of the trap (~28 µm). The length of *S. pombe* cells typically varied between 7 and 14 µm, which is well in the linear range of the EIS system.

The acquired signal spectra over the whole frequency range in the impedance measurements show maximal differences of the relative magnitude at 1 MHz, and conspicuous differences of the relative phase above 5 MHz (**Supplementary Figs. S5−7**). The stacked beads, especially those with the same diameter, are difficult to distinguish in bright-field micrographs. In contrast, the EIS results (**Supplementary Fig. S3d−f**) show a clear classification of all the arrangements of measured beads using A_r at 1 MHz and θ_r at 5 MHz. Even stacked beads of 6+8 µm and 7+7 µm – with only a slight difference in the obstructed area of the orifice and overall height – can be differentiated by using a two-sample *t*-test (**Supplementary Fig. S3f** insert).

Variations in the impedance of empty traps were not observed during the duration of the experiments (up to 3 hours). It is important to note that θ_r at 5 MHz shows a rigorously linear correlation to A_r at 1 MHz, which can be expected for invariable plastic beads. Moreover, the

results of our impedance measurements of single 6-, 7-, and 8-μm-diameter beads show a somewhat lower relative coefficient of variation (CV of ~0.045%) in comparison to the provided datasheet (CV of ~1.3%) based on Coulter counter measurements (**Supplementary Table S2**).

Supplementary Note S2: Parameters for modeling

The geometric and electrical parameters of the *S. pombe* cell for the modeling (**Supplementary Table S3**) were chosen according as follows: Diameters of cell and nucleus were measured from the confocal micrographs. The thickness of cell wall, plasma membrane, and nuclear membrane, and the conductivity of plasma membrane and nuclear membrane were values from literature [1]. The conductivity of the used medium was measured by using a Mycom CLM 141 conductivity transmitter (Endress+Hauser, Switzerland) combined with a JUMO Blackline conductivity probe (JUMO, Switzerland). The conductivity of the cell wall is correlated to the conductivity of suspension medium, and was set to 0.053 S/m, one tenth of the medium conductivity. The conductivity of the nucleus was set to 1.0 S/m according to literature [2]. The relative permittivities of cell wall, plasma membrane, cytosome, nucleus, and nuclear membrane were estimated based on the literature values [1,2]. The relative permittivity of the used medium was set to 80.

Reference:

[1] Talary, M. S., Burt, J. P. H., Tame, J. A., & Pethig, R. Electromanipulation and separation of cells using travelling electric fields. *J. Phys. D-Appl. Phys.* **29**, 2198-2203 (1996).

[2] Kriegmaier, M., Zimmermann, M., Wolf, K., Zimmermann, U., & Sukhorukov, V. L. Dielectric spectroscopy of Schizosaccharomyces pombe using electrorotation and electroorientation. *Biochimica et Biophysica Acta (BBA) - General Subjects*, **1568**, 135-146 (2001).
